# Automated Real-Time Tool for Promoting Crisis Resource Use for Suicide Risk (ResourceBot): Development and Usability Study

**DOI:** 10.2196/58409

**Published:** 2024-10-31

**Authors:** Daniel DL Coppersmith, Kate H Bentley, Evan M Kleiman, Adam C Jaroszewski, Merryn Daniel, Matthew K Nock

**Affiliations:** 1 Department of Psychology Harvard University Cambridge, MA United States; 2 Department of Psychiatry Massachusetts General Hospital/Harvard Medical School Boston, MA United States; 3 Department of Psychology Rutgers, The State University of New Jersey Piscataway, NJ United States; 4 Franciscan Children's Hospital Brighton, MA United States

**Keywords:** suicidal thoughts, suicidal behaviors, ecological momentary assessment, crisis resources, real-time tool, self-report, psychoeducation, app

## Abstract

**Background:**

Real-time monitoring captures information about suicidal thoughts and behaviors (STBs) as they occur and offers great promise to learn about STBs. However, this approach also introduces questions about how to monitor and respond to real-time information about STBs. Given the increasing use of real-time monitoring, there is a need for novel, effective, and scalable tools for responding to suicide risk in real time.

**Objective:**

The goal of this study was to develop and test an automated tool (ResourceBot) that promotes the use of crisis services (eg, 988) in real time through a rule-based (ie, if-then) brief barrier reduction intervention.

**Methods:**

ResourceBot was tested in a 2-week real-time monitoring study of 74 adults with recent suicidal thoughts.

**Results:**

ResourceBot was deployed 221 times to 36 participants. There was high engagement with ResourceBot (ie, 87% of the time ResourceBot was deployed, a participant opened the tool and submitted a response to it), but zero participants reported using crisis services after engaging with ResourceBot. The most reported reasons for not using crisis services were beliefs that the resources would not help, wanting to handle things on one’s own, and the resources requiring too much time or effort. At the end of the study, participants rated ResourceBot with good usability (mean of 75.6 out of 100) and satisfaction (mean of 20.8 out of 32).

**Conclusions:**

This study highlights both the possibilities and challenges of developing effective real-time interventions for suicide risk and areas for refinement in future work.

## Introduction

### Overview

Real-time monitoring methods—such as ecological momentary assessment (EMA)—capture fine-grained, “real-world” information about suicidal thoughts and behaviors (STBs) as they occur and thus have immense potential to advance our understanding of suicide [1,2]. The promise of real-time monitoring methods for STBs has been widely recognized, as evidenced in part by the recent proliferation of published studies using EMA to study STBs. A recent systematic review identified 45 articles that have used real-time monitoring methods to study STBs [3].

Collecting information about STBs in real time, however, poses important safety, ethical, and methodological concerns [4]. One complex ethical challenge is regarding how to monitor or respond to incoming information about STBs from suicidal or self-injuring individuals. For example, when participants submit a survey response indicating current suicidal intent that researchers can access in real time, should the study team intervene? How should the study team determine when an intervention is needed? What should the intervention involve?

A consensus statement (generated from a panel of 24 experts) on the ethical and safety practices for conducting real-time monitoring studies of individuals at risk for suicide and related behaviors was recently released [4]. There was a strong (about 94%) agreement that when participants provide a “high-risk” response, the study team should reach out to them directly to conduct a suicide risk assessment as soon as possible (within 12-24 hours for responses indicating “imminent” risk). An exception the panel noted, however, was anonymous studies where contact information for participants is not known. A systematic review of practices in 59 previous or ongoing digital monitoring studies of STBs [5], however, indicates a gap between this apparent consensus and reality, as just over half (58%) of studies reported monitoring and intervening upon incoming responses during the study. Thus, there remains a notable departure between expert consensus and real-world practices for responding to incoming data. The other most common safety practice identified in this review was automated notifications (eg, pop-up messages with crisis resources) triggered by specific survey responses, which was used in roughly half of the studies included.

Both common approaches of researchers intervening, and static pop-up messages have significant limitations. Static messages are easy for participants to habituate to or ignore, especially during periods of high distress. Human- (often clinician-led) active interventions (eg, calling participants) by the research team are resource-intensive and have the potential to cause undesired reactivity. If participants are aware the researchers will act if they provide a “high-risk” response, participants may underreport STBs (or stop responding to study surveys entirely) to avoid an unwanted intervention. A recent empirical investigation of this issue of reactivity found mixed support for reactivity to real-time interventions (in this case, phone outreach by the study team) [6]. If responding to incoming data on STBs does influence individuals’ responding behavior, this could muddle the validity of the resultant study data. Another key limitation is the feasibility of monitoring and responding to incoming data, as this approach tends to require considerable staff, technology systems, and funding. The resources required for these safety protocols may partially explain the gap between expert consensus and real-world practices.

Given the increasing use of real-time monitoring methods [7], there is a need for novel, effective, and scalable tools for responding to suicide risk in real time. Recent advances in mobile technologies have the potential to facilitate automated, potentially highly efficient risk assessment strategies (ie, that do not require a clinician calling and may be less subject to reactivity) and deployment of specific types of notifications or alerts delivered directly to participants. Automated assessments and interventions could be faster and less burdensome for both participants and researchers. Automated tools (here, referring to systems that use rule-based [eg, if-then] logic, not those that use generative artificial intelligence; Blease and Torous [8]) might be more effective than human interventions because they can reach the participant faster than study staff. Recently developed rule-based automated assessments and interventions for STBs have shown promise. One such automated intervention consists of a brief, automated risk assessment and barrier reduction intervention (BRI) designed to increase the use of crisis resources [9]. The BRI component includes psychoeducation designed to reduce perceived barriers to using crisis resources by clearing up misconceptions on which these barriers may be based. A large-scale clinical trial found that this intervention was associated with a 23% increase in the use of crisis services [9]. A similar trial of an automated intervention also found that a brief automated intervention could increase the reported use of crisis resources [10]. The promise of this type of intervention for real-time monitoring is that it could guide participants to resources during high-risk situations. This type of intervention is consistent with recent calls for just-in-time adaptive interventions for suicide prevention, which are intended to provide the right type of support at the right time [11,12]. Therefore, providing ethical, scalable, and fast risk management during research studies.

This automated intervention, however, has only been tested at a single time point [9,10] and has never been tested in the context of participation in real time, longitudinal monitoring of those at risk for suicide. Understanding how this tool translates to this context is crucial given that one cannot assume engagement and efficacy generalize across contexts in digital health [13]. Engagement is a crucial first step given that digital interventions often suffer from low engagement and a quick drop-off in use over time. In short, to realize the potential of an automated digital intervention, participants must engage with the intervention. Therefore, it is crucial to first adapt this intervention for real-time monitoring. Second, it is important to understand the feasibility, acceptability, and utility of the intervention for real-time monitoring. The development of such a tool has the potential to improve the safety, scale, and breadth of real-time monitoring studies of STBs.

### Study Aims and Outcomes

The overall project aims were (1) to adapt an evidence-based BRI that aims to increase the use of crisis resources for deployment in real time monitoring research and to (2) to test the feasibility, acceptability, and utility of this tool in a real-time monitoring study of suicidal thoughts. For the latter objective, the key outcomes of interest were as follows: (1) Do people engage with the BRI? (2) Do people contact crisis resources after the BRI? (3) What do people report about the acceptability and usability of the BRI?

## Methods

### Adapting BRI

**Figure 1:**
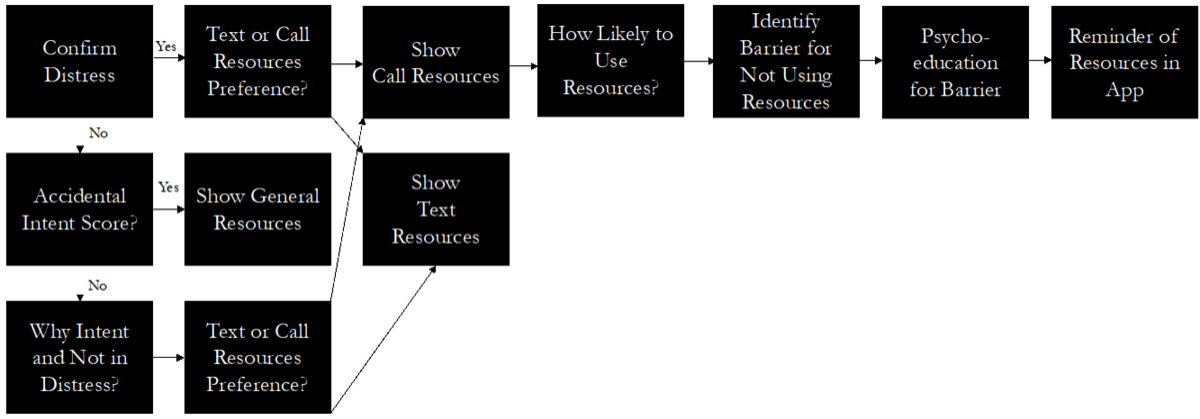
General overview of ResourceBot flow.

**Figure 2:**
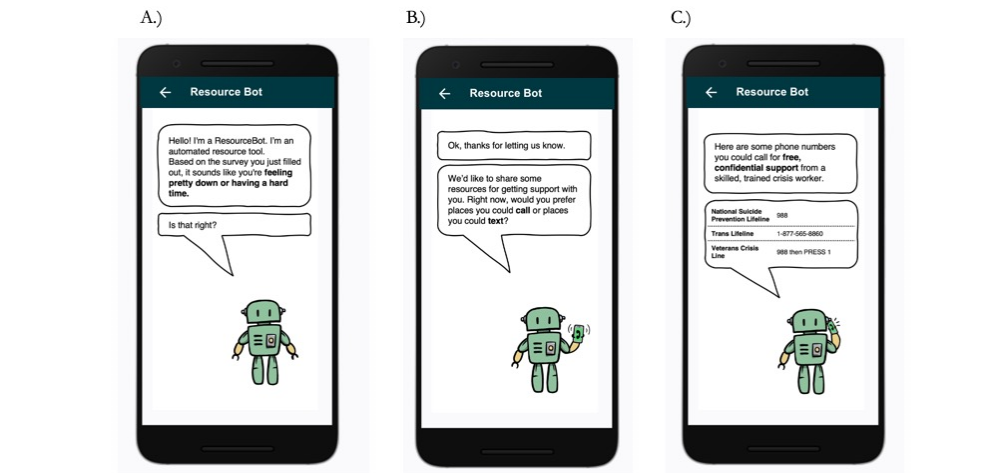
Example of ResourceBot offering resources. (A) Confirming current distress. (B) Asking about the type of resources to view. (C) Providing resources to call if participants selected that they wanted resources to call.

**Figure 3:**
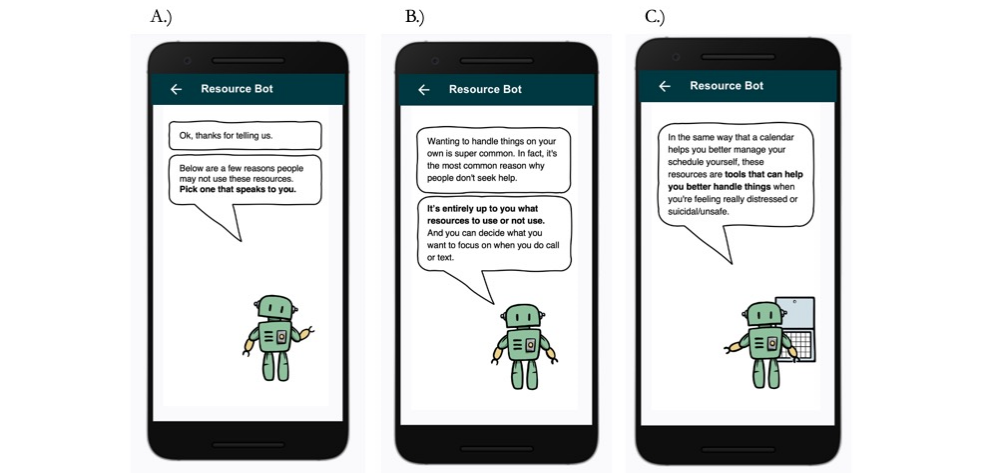
Barrier reduction intervention in ResourceBot. (A) Asking participants to select barriers to using resources. (B) The first psychoeducation slide shows if “I can handle it on my own” is selected. (C) The second psychoeducation slide shows if “I can handle it on my own” is selected.

To adapt the BRI [9], members of the author team first met to develop the workflow of the intervention as well as the text to be deployed. Over multiple meetings, the author team iterated on the workflow to be appropriate for the context of a real-time monitoring study. The main changes to be appropriate for real-time monitoring research included changing the beginning of the workflow to focus on the recent submission of a survey with self-report suicidal intent and for the text to be brief enough for viewing on mobile phones. The authors then worked with a graphic designer (MD) to name the BRI and develop images to pair with each text screen of the intervention. Images were added to promote engagement and to help differentiate the BRI from the base real-time survey questions. The BRI was named ResourceBot and images of the ResourceBot were generated for each screen. In total, there were 23 unique images or text slides generated. The ResourceBot was designed to be triggered after a participant submits a survey with elevated suicidal intent. The workflow of ResourceBot is: (1) confirm current distress (to ensure the participant-reported distress was not made in error), (2) offer resources, (3) identify barriers to using resources, and (4) provide psychoeducation to promote resource use. A general overview of the ResourceBot workflow is provided in Figure 1 and example slides of the tool are provided in Figures 2 and 3. All images of the ResourceBot and the decision logic are provided in Multimedia Appendix 1. ResourceBot was built and deployed directly in the Metricwire app (Metricwire Inc), which was also used for real-time monitoring surveys.

As part of the development of the ResourceBot tool, we ran a pilot study with 8 participants to primarily determine when to trigger ResourceBot among other topics. In this pilot study, across 369 completed total surveys, the ResourceBot tool was only deployed twice to one participant. This one participant engaged with the ResourceBot and the crisis resources it provided. Based on the results of this pilot study, we lowered the threshold for triggering the ResourceBot from a suicidal intent rating on the EMA or daily survey of greater than 3 (out of 10) to greater than 1 (out of 10) for the main wave of data collection. This threshold was lowered so that a greater number of participants would be provided with the tool and able to provide feedback on it. Given that in the pilot, we found that the ResourceBot was successfully deployed, we proceeded to the main wave of data collection.

### Participants

Participants were 74 adults who were recruited through the Prolific research platform. Prolific was selected for recruitment given it has been associated with high-quality data [14,15]. The demographics and clinical characteristics of the participants are provided in Table 1. The specific inclusion criteria for the study were suicidal thoughts in the past week, the ability to speak and write English fluently, access to an internet-capable smartphone, and living in the United States. To identify participants eligible for the study, a screening survey study was sent out to participants on the platform who lived in the United States, were fluent in English, had at least a 90% approval rating of past studies on Prolific, and endorsed a lifetime history of mental illness. We used the filter of lifetime history of mental illness to increase the prevalence of suicidal thinking in the population initially screened for recent suicidal thoughts. Consistent with recommendations for web-based data collection [16] and to help ensure quality data and attentive responding, suicidal thoughts were asked about in multiple ways on the screening survey (eg, binary lifetime presence of thoughts, ordinal recency of thoughts, and text entry number of days with thoughts). To be included participants needed to provide a consistent response pattern on the screening survey of endorsing the lifetime presence of suicidal thoughts, reporting the most recent time they had suicidal thoughts in the past week, and writing a number greater than zero for lifetime days with suicidal thoughts. Participants were compensated US $0.60 for completing the brief screening survey. Participants who met eligibility criteria based on the screening survey were then invited to the main study. To be included in the current analysis, participants had to complete at least 1 real-time survey.

**Table 1:** Participant demographics and clinical variables (n=74).

Demographics			Variables
Age (years), mean (range)			34.7 (20 to 62)
**Sex assigned at birth, n**			
	Female	37	
	Male	37	
**Gender identity, n**			
	Female	31	
	Male	36	
	Genderqueer, non-binary, gender fluid	6	
	Other	1	
**Race, n**			
	White	59	
	Black	6	
	Asian	3	
	Multiracial	5	
	Other	1	
**Ethnicity, n**			
	Hispanic/Latino	7	
**Highest education level, n**			
	Less than high school	1	
	High school/GED^a^	11	
	Some college	22	
	2-year college degree	6	
	4-year college degree	30	
	Master degree	3	
	Professional degree	1	
**Lifetime suicide attempt, n**			
	Yes	46	
Lifetime days with suicidal thoughts, median (SD)			550 (2451)
Patient Health Questionnaire-9, mean (SD)			18.0 (6.6)
Generalized Anxiety Disorder-7, mean (SD)			14.2 (5.6)
**Psychotherapy history, n**			
	Lifetime use	59	
	Current use	22	
**Medication for mental health history, n**			
	Lifetime use	59	
	Current use	29	

^a^GED: General Education Development.

### Ethical Considerations

All study procedures were approved by the Harvard University Area institutional review board (protocol IRB22-0012; “Automated Real Time Safety and Monitoring Study”). All participants provided informed consent. Following the screening survey, eligible participants were sent the consent form and baseline survey. The informed consent form contained specific language about how real-time responses were not being monitored by the study team and automated messages would encourage resources.

### Procedure

In the baseline survey, participants completed questionnaires assessing STBs, psychiatric symptoms, and mental health care history. At the end of the survey, participants were provided with instructions for downloading the Metricwire smartphone app and then confirmed that they had downloaded the app before submitting the baseline survey for approval. Participants were compensated US $10 for completing the baseline survey. Participants were then sent to the Prolific platform, an anonymous login for the Metricwire smartphone app.

The real-time monitoring period was 2 weeks long and began the day after participants logged into their anonymous accounts. Six surveys were sent each day. Five momentary surveys were sent semirandomly between 9 AM and 9 PM and spaced at least 90 minutes apart. One daily survey was sent at 8 PM each day. The momentary survey stayed open to complete for 1 hour and the daily survey for 2 hours. The last momentary survey of the day and the daily survey could overlap; ultimately, 11% percent of momentary surveys were submitted during the hours of 8 PM to 10 PM (after the daily survey was prompted). Participants were paid US $0.25 for each survey they completed. If participants completed 5 or more surveys in a day, they received a US $1.00 bonus for that day. All payments for the real-time surveys were sent the day after the real-time monitoring period was complete.

On the day after the last day of the real-time monitoring period, participants were sent an exit survey via MetricWire asking them about their experiences in the study. If a participant reported that they received the ResourceBot, they were then asked questions about the acceptability and usability of it. The exit survey stayed open to complete for 8 hours. For completing the exit survey, participants were paid US $3. With this payment structure, participants could earn up to US $48 in the study. All payments were sent through Prolific.

### Baseline Assessment Surveys

In the baseline assessment, participants completed a self-report version of the Self-Injurious Thoughts and Behaviors Interview—Revised (SITBI-R; Fox et al [17]). The SITBI-R measures the presence, frequency, recency, and other aspects of self-injurious thoughts and behaviors. The SITBI-R has shown excellent reliability and validity [17]. Participants also completed the Patient Health Questionnaire-9 (PHQ-9; Kroenke et al [18]). The PHQ-9 is a widely used brief measure of the severity of symptoms of depression in the past 2 weeks. PHQ-9 scores range from 0 to 27. In this study, the PHQ-9 had excellent internal consistency (Cronbach α=0.89). The Generalized Anxiety Disorder 7-item (GAD-7; Spitzer et al [19]) was also administered at baseline. The GAD-7 is a brief measure of the severity of symptoms of anxiety in the past 2 weeks. GAD-7 scores range from 0 to 21. In this study, the GAD-7 had excellent internal consistency (Cronbach α=0.90).

We also measured participants’ mental health treatment history and crisis service use with measures created for this study. For mental health history, participants were asked if they had ever received any form of mental health treatment from a professional. If so, they were asked if they had ever received talk therapy for mental health and if they were currently receiving talk therapy. Participants were also asked if they had ever been prescribed medications for mental health reasons and if they were currently being prescribed medications for mental health reasons. For crisis resources use, participants were asked if they had “ever called a suicide crisis lifeline (eg, 988 Suicide & Crisis Lifeline)” and if they had ever “texted a suicide crisis service (eg, Crisis Text Line).” If a participant endorsed using a crisis resource, they were asked how helpful it was on a 0 (not at all helpful) to 5 (very helpful) scale and how likely they were to use the resource in the future on a 0 (not at all likely) to 5 (very likely) scale. All participants were also asked, “Below are a few reasons that people may not call or text suicide crisis services. Do any of these speak to you as reasons why you wouldn't call or text a suicide crisis service in the future?” The reasons listed were: “I can handle things on my own,” “Too much time/effort,” “No professionals,” “No police,” “They won’t help,” and “None of these reasons.”

### EMA Items

The momentary and daily surveys contained multiple items on suicidal thinking, affective states, and cognitive processes. For the present analyses, the most relevant item is the suicidal intent item, which was used to trigger the ResourceBot. The exact item wording was “How strong is your intent to kill yourself right now? Intent = to what extent are you actually going to kill yourself.” Participants rated this item on a 0 (“not at all”) to 10 (“very strong”) scale. This item has been used in previous real-time studies [20,21] and a similar item has shown predictive validity for suicidal behavior [22]. A daily version of the item was also included, “Today how strong was your intent to kill yourself? Intent = to what extent are you actually going to kill yourself.” The daily item had the same scale and anchors as the momentary item. Suicidal intent was selected to trigger the ResourceBot because, in a consensus statement, it was identified as a key piece of information for determining real-time risk [4]. Furthermore, the level of suicidal intent has been used to determine interventions in other real-time risk protocols [5,6].

Another relevant item in the daily survey was an item on crisis resource use. Participants were specifically asked, “Today, did you use any crisis resources? For example, did you call 988?” with the response options of yes or no. If a participant selected yes, then a participant would be asked “What crisis resource did you use? For example, calling 988, texting crisis text line.” If a participant selected yes, they would also be asked how helpful the resource was. These items were included to capture additional data on crisis resource use in case participants did not complete the ResourceBot follow-up survey.

### Crisis Resources

At all times in the real-time monitoring period, there was an always-available list of resources they could open (ie, “resources survey”) in the MetricWire app. The resources survey contained a list of the following resources: National Suicide Prevention Lifeline, Crisis Text Line, Trans Lifeline, Veterans Crisis Line Chat, Veterans Crisis Line, and Trevor Project Chat. A participant could select a resource from the list, which would take them to a page with more information on the resource and how to contact them. The page included a direct link to the webpage of the resource. Participants were told that “the research team may be able to see if you select a resource, but the team cannot see what you communicate or share with the support lines.” These resources were selected because they offered support through different means of communication (eg, calling or texting) and support for different populations. Additionally, at the end of all real-time surveys, participants were reminded that the National Suicide Prevention Lifeline, Crisis Text Line, and their local emergency department were helpful resources.

### ResourceBot Tool

The ResourceBot tool was built directly in MetricWire. When a participant submitted a survey with a suicidal intent rating greater than 1, it would trigger the deployment of the ResourceBot tool. This threshold of suicidal intent was lower than the threshold used in previous studies [5] because the intervention is lower intensity compared with other interventions (eg, clinician contact). A stop rule in place was that the ResourceBot tool would be only deployed once a day to limit the burden and increase engagement. The ResourceBot tool was sent immediately after the survey submission. If a participant did not open the ResourceBot survey, then a reminder notification was sent 5 minutes after the initial deployment. The ResourceBot survey stayed open for 4 hours. Once a participant opened the ResourceBot survey, a participant was guided through a protocol that (1) confirmed current distress, (2) offered crisis resources, (3) identified barriers to crisis resource use, and (4) provided psychosocial education on resources. An example interaction is provided in Figures 2 and 3. The tool is designed to overcome common concerns and misconceptions (ie, barriers) related to using crisis services, thereby increasing the use of these services.

### ResourceBot Follow-Up Survey

One hour after the ResourceBot survey was submitted, a brief follow-up survey was sent. The survey asked if a participant used a resource since they were sent the ResourceBot. If yes, they were asked what resource they used and how helpful the resource was. If not, participants were asked why they did not use a resource. The response options for why they did not use a resource included: “Too much time/effort,” “Didn’t think it would help,” “Felt better without them,” “I handled it on my own,” and “Other.” Participants could enter more information into an open-ended text field if they selected “Other.”

### Exit Survey

At the end of the EMA period, participants were sent a survey asking them about their experiences in the study. All participants were sent questions about the overall study and participants who were sent the ResourceBot were sent additional questions about the ResourceBot. All participants were asked, “Did you feel comfortable answering the cellphone questions honestly?” and rated it on a scale of 0 (not at all) to 5 (very much). All participants were asked, “Did you receive the ResourceBot, which directed you to crisis services, in the study?” and shown a picture of the ResourceBot as a reminder. If a participant endorsed receiving the ResourceBot, they were sent the Client Satisfaction Questionnaire (CSQ; Larsen et al [23]) and the System Usability Scale (SUS; Lewis and Sauro [24]). The CSQ is an 8-item scale that produces a score from 8 to 32 with higher scores indicating greater satisfaction. In this study, the CSQ was used as a measure of acceptability and had excellent internal consistency (Cronbach α=0.96) The SUS is a 10-item scale that produces an original score of 0 to 40 which is then multiplied by 2.5 to create scaled scores of 0 to 100 with higher scores indicating that the user rates the system as more useable. In this study, the SUS had excellent internal consistency (Cronbach α=0.91).

### Statistical Analysis

For all analyses, we focus on descriptive statistics. For the first aim of whether people engage with the ResourceBot, we focus on how often participants open and submit the ResourceBot survey. Although there are multiple ways to operationalize engagement [25], we highlight this simple definition of engagement for this first examination of ResourceBot. We also report on data provided within the ResourceBot survey, including the endorsed barriers to using crisis resources.

One factor that could have affected participants’ engagement with the ResourceBot is the current level of suicidal intent. For example, a participant with higher levels of current intent (eg, 9 out of 10) and possibly greater risk may engage with the tool in a different way than a participant with lower levels of current intent (eg, 2 out of 10). To understand the relationship between the level of intent prior to the ResourceBot and engagement with the ResourceBot we ran additional analyses. We identified the momentary survey submitted closest in time (ie, the trigger survey) to the submitted ResourceBot survey. This resulted in momentary intent ratings for 181 of the 192 ResourceBot engagements; the 11 other engagements were triggered by a daily survey report. We focused on the 181 engagements for the subsequent descriptive analyses. The average time difference between the submitted momentary survey and the submitted ResourceBot survey was 7.6 minutes. We then categorized the momentary intent levels into low and high levels. We operationalized low as a score of 2, 3, or 4 and high as a score of greater than 5. This resulted in 92 low-intent ResourceBot engagements and 89 high-intent ResourceBot engagements. We present descriptive statistics on data within the ResourceBot encounter by momentary intent level.

Due to the potential for habituation to ResourceBot content with multiple deployments over time, we also isolated each participant’s first submitted engagement with the ResourceBot and presented descriptive statistics on data within this first encounter with the ResourceBot.

For the second aim, if people contact crisis resources after engaging with ResourceBot, we focus on how often in the ResourceBot follow-up survey do people report using crisis resources. We also report on crisis resource use reported in the daily survey as well as the frequency of viewing the crisis resources. For the third aim, we report on the descriptive statistics on exit survey scores on acceptability and usability. We also report additional exit survey data on the honesty of responding. Together these analyses use multiple sources to comprehensively describe the feasibility, acceptability, and utility of a real-time crisis resource tool. All data analysis codes and results can be viewed on the Open Science Framework [26].

## Results

### Descriptive Statistics

Baseline data on lifetime use of crisis hotlines, experiences on crisis hotlines, and barriers to future use of crisis hotlines are provided in Table 2. Most participants (49/74, 66% for calling and 58/74, 78% for texting) had not used crisis hotlines in their life. Participants who had previously used crisis hotlines reported that they in general were not helpful (calling mean helpfulness=1.12 out of 5; texting mean helpfulness=1.19 out of 5). Participants reported on average they were not very likely to use hotlines in the future. The most frequently endorsed reason for not using crisis hotlines in the future was the belief that they would not help.

**Table 2:** Baseline crisis hotline lifetime histories.

Baseline histories		Variables
**Lifetime called crisis line, n (%)**		
	Yes (Percent)	25 (34)
**Lifetime texted crisis line, n (%)**		
	Yes (Percent)	16 (22)
Helpfulness of calling crisis line^a^, mean (SD)		1.12 (1.54)
Helpfulness of texting call line^b^, mean (SD)		1.19 (1.33)
How likely to call crisis line in the future^c^, mean (SD)		1.14 (1.47)
How likely to text crisis line in the future^d^, mean (SD)		1.35 (1.62)
**Reasons for not using crisis lines in the future^e^**		
	I can handle things on my own	26
	Too much time or effort	12
	No professionals	12
	No police	17
	They would not help	49
	None of these reasons	6

^a^Only answered by participants who answered they had called a crisis line (n=25).

^b^Only answered by participants who answered they had texted a crisis line (n=16).

^c^Answered by all participants (n=74).

^d^Answered by all participants (n=74).

^e^Participants could select multiple reasons.

**Figure 4:**
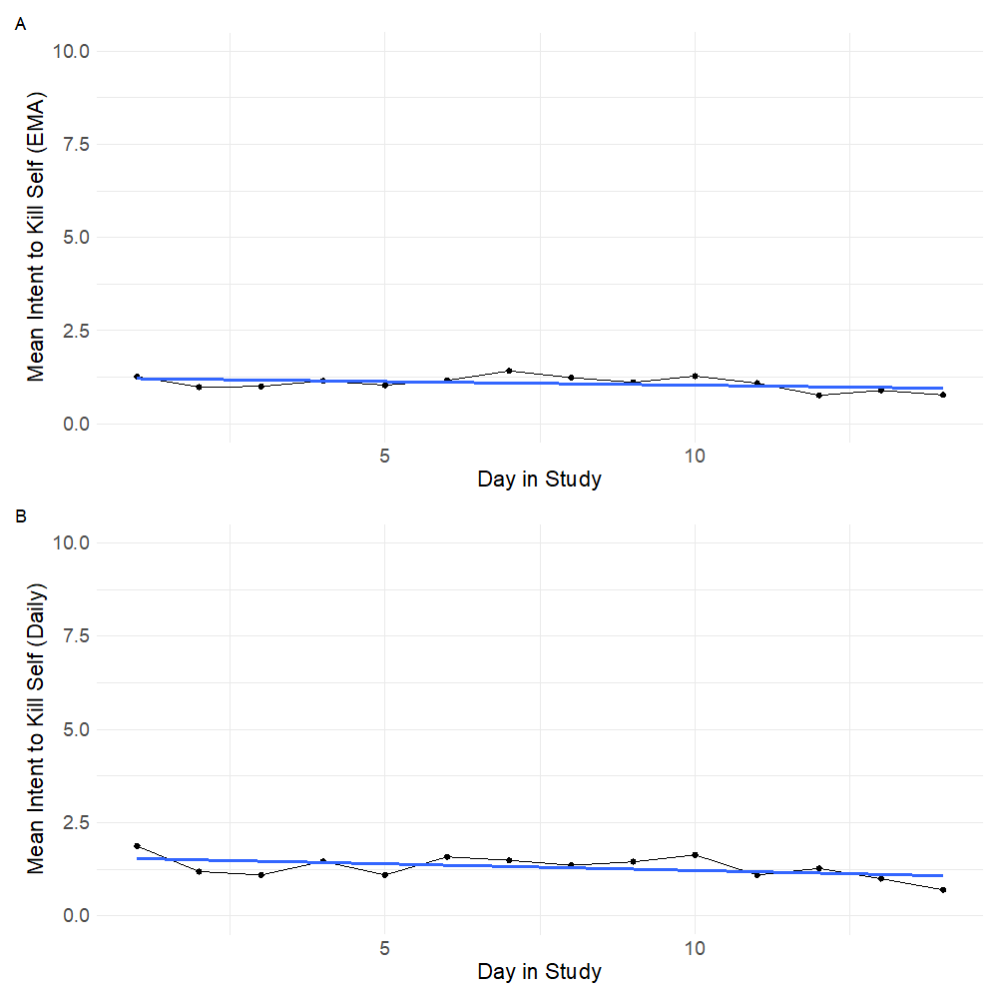
Mean suicidal intent scores over time in the study. (A) Mean EMA intent scores by day in study. (B) Mean daily intent scores by day in study. Blue line is the linear trend of the mean intent score by day in the study. EMA: ecological momentary assessment.

Participants completed 2909 momentary surveys and 679 daily surveys. A total of 74 participants completed at least 1 momentary survey and 72 participants completed at least 1 daily survey. The average number of momentary surveys submitted was 39.3 (range 2 to 70) and for daily surveys, it was 9.4 (range 1 to 14). The average compliance rate for the momentary surveys was 56% (range 3% to 100%) and for the daily surveys was 67% (range 7% to 100%). The daily averages of EMA and Daily survey scores of the intent to kill oneself item are shown in Figure 4.

The ResourceBot was deployed 221 times to 36 participants. A total of 35 participants engaged with the ResourceBot at least once. The ResourceBot was deployed and engaged multiple times by 28 (80%) of the 35 participants who engaged it at least once. The other 7 (20%) of the 35 participants engaged with it only once.

The exit survey was completed by 44 participants. We compared participants who completed the exit survey (n=44) to those who did not (n=30) on EMA compliance percentage, daily survey compliance percentage, mean EMA suicidal intent severity, and mean daily suicidal intent severity. We conducted this descriptive retention analysis to understand if the type of participant who completed the exit survey may be biased in some way. We found that participants who completed the exit survey had higher EMA (72% vs 34%) and daily survey (81% vs 46%) compliance rates than those who did not complete the exit survey. We also found similar mean EMA (1.31 completers vs 1.15 noncompleters) and daily suicidal intent ratings (1.48 completers vs 1.43 noncompleters) by exit survey status.

Among those who completed the exit survey, 19 reported receiving the ResourceBot. We cross-checked participants’ self-reports of receiving the ResourceBot with the ResourceBot deployment data. Eighteen of the 19 who reported receiving the ResourceBot in the exit survey matched with the ResourceBot deployment data. The one participant who reported receiving the ResourceBot, but did not actually receive it was excluded from the ResourceBot exit survey analysis. One of the 18 participants did not complete all items for the CSQ and therefore, we report on 17 participants for the CSQ.

### Do People Engage With the ResourceBot?

There was 87% (192 out of 221) overall engagement (defined as opening and submitting) with the ResourceBot tool and 86% (165 out of 192) compliance with the ResourceBot follow-up survey. The different components of the ResourceBot and the frequency of responses are provided in Table 3. In the majority of deployments (122 out of 192) participants confirmed that they were in distress. For participants who reported that they were not in distress, the most commonly reported reason was being used for these thoughts or feelings. Text resources (n=81) were more frequently selected than call resources (n=19). For the likelihood of using resources, the most common response was not likely (n=48). For barriers to using resources, the most common responses included that “it won’t help” and “I can handle it on my own.” As shown in Table 3, participants often skipped out of the ResourceBot at various stages of the tool.

**Table 3:** ResourceBot responses for all ResourceBot engagements.

Question	Response (n)
It sounds like you’re feeling pretty down or having a hard time. Is that right? (n=192)	Yes (122) No (14) Skipped (56)
Why are you having high intent but not feeling down/having a hard time? (n=8)	I am used to these thoughts/feelings (7) I don’t need help for these thoughts/feelings (1)
Prefer places could call or places you could text? (n=130)	Call (19) Text (81) Skipped (30)
How likely to use the resources shared? (n=100)	Not Likely (48) Somewhat Likely (34) Very Likely (6) Skipped (12)
Reasons people may not use these resources (n=82; shown if not likely or somewhat likely to use resources)	It won’t help (37) I can handle it on my own (26) Too much time/effort (5) No police (5) I may not use these resources for a reason not otherwise listed (4) No professionals (3) Skipped (2)
Reasons people may not use these resources (n=6; shown if very likely to use resources)	It won’t help (1) I can handle it on my own (2) Too much time/effort (0) No police (0) Not really - another reason (3) No professionals (0) Skipped (0)

Responses to the ResourceBot by level of momentary intent are presented in Table 4. The patterns of responding were similar across low and high levels of intent. For example, across both low and high levels of intent, participants most commonly reported being not likely to use the Resources presented and most frequently endorsed the barrier of the belief that the resources would not help. Results from the first encounter with ResourceBot only are presented in Table 5. Results from the first encounters showed similar trends to data from all encounters.

**Table 4:** ResourceBot responses by ecological momentary assessment suicidal intent severity (n=182).

Question	Low Intent Responses (n)	High Intent Responses (n)
It sounds like you’re feeling pretty down or having a hard time. Is that right?	Yes (53) No (8) Skipped (32)	Yes (64) No (6) Skipped (19)
Why are you having high intent but not feeling down/having a hard time?	I am used to these thoughts/feelings (5) I don’t need help for these thoughts/feelings (0)	I am used to these thoughts/feelings (2) I don’t need help for these thoughts/feelings (1)
Prefer places could call or places you could text?	Call (7) Text (41) Skipped (10)	Call (12) Text (36) Skipped (17)
How likely to use the resources shared?	Not Likely (21) Somewhat Likely (18) Very Likely (2) Skipped (7)	Not Likely (27) Somewhat Likely (12) Very Likely (4) Skipped (5)
Reasons people may not use these resources	It won’t help (15) I can handle it on my own (15) Too much time/effort (3) No police (3) I may not use these resources for a reason not otherwise listed (2) No professionals (0) Skipped (1)	It won’t help (21) I can handle it on my own (9) Too much time/effort (2) No police (2) I may not use these resources for a reason not otherwise listed (2) No professionals (3) Skipped (0)
Reasons people may not use these resources	It won’t help (0) I can handle it on my own (2) Too much time/effort (0) No police (0) Not really - another reason (0) No professionals (0) Skipped (0)	It won’t help (1) I can handle it on my own (0) Too much time/effort (0) No police (0) Not really - another reason (3) No professionals (0) Skipped (0)

**Table 5:** ResourceBot responses (first engagement only).

Question	Response (n)
It sounds like you’re feeling pretty down or having a hard time. Is that right? (n=35)	Yes (16) No (0) Skipped (19)
Prefer places could call or places you could text? (n=16)	Call (1) Text (12) Skipped (3)
How likely to use the resources shared? (n=13)	Not Likely (4) Somewhat Likely (5) Very Likely (0) Skipped (4)
Reasons people may not use these resources (n=9)	It won’t help (5) I can handle it on my own (2) Too much time/effort (0) No police (1) I may not use these resources for a reason not otherwise listed (1) No professionals (0) Skipped (0)

### Do People Contact Crisis Resources After Engaging With ResourceBot?

In the ResourceBot follow-up survey, 0 participants reported using crisis resources. Participants could endorse multiple reasons for not using resources in the follow-up survey. The frequency of the reasons for not using the resources was as follows: did not think it would help (76/165, 46%), I handled it on my own (70/165, 42%), too much time and effort (33/165, 20%), felt better without them (24/165, 15%), and other (5/165, 3%). Two times participants did not answer this question.

In the daily survey, there were 3 total times participants reported using crisis resources that day from 3 separate participants. One participant reported calling a crisis line, one reported attending group therapy, and one did not remember the exact resource they used. The helpfulness ratings for 3 instances were 0, 4, and 5 (out of 5).

**Figure 5:**
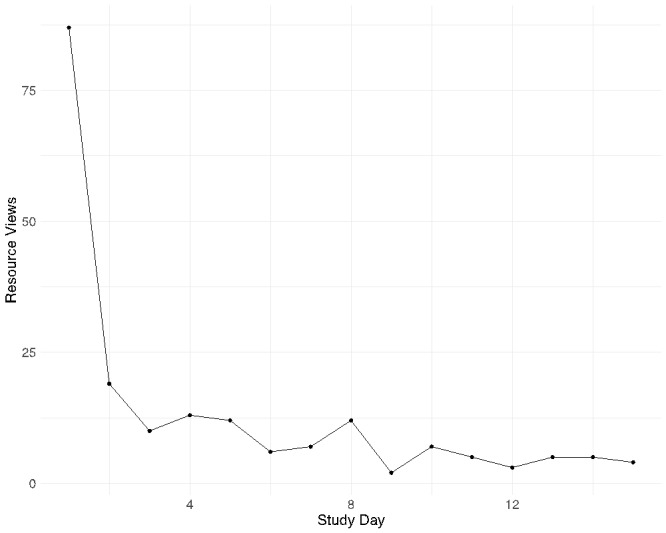
Number of views of resources by day in study. Study day 1 refers to the day a participant first logged into the Metricwire smartphone app and day 2 refers to the first day of smartphone surveys.

The crisis resources survey (ie, the constantly available list of resources that participants could open) was opened and submitted a total of 312 times in the survey across 59 participants. A total of 113 times participants opened the survey and skipped out without selecting a resource to view, we therefore report on the remaining 199 times participants selected a resource to view. The frequency of viewing by time in the study is shown in Figure 4. In Figure 4, day 1 refers to the day a participant first logged into the Metricwire smartphone app and day 2 refers to the first day of smartphone surveys. The frequency of viewing for each type of resource was the following (Figure 5): Crisis Text Line (69/197, 35%), National Suicide Prevention Lifeline (58/197, 29%), Trevor Project Chat (29/197, 15%), Trans Lifeline (17/197, 9%), Veteran’s Crisis Line Chat (13/197, 6%), and Veteran’s Crisis Line (13/197, 6%).

### What Do People Report About the Acceptability and Usability of ResourceBot?

The mean for the CSQ was 21 (out of 32) and the SD was 5.96. The mean for the SUS was 76.7 (out of 100) and the SD was 17.06. The mean and SD for all items on both scales are provided in Multimedia Appendix 1. For the comfort with answering questions honestly item, the average rating was 4.30 (out of 5).

## Discussion

The aims of the current project were to adapt an evidence-based BRI into a new tool for smartphone-based delivery (ResourceBot) that aims to increase the use of crisis resources and to test the feasibility, acceptability, and utility of this tool in real time monitoring. There were 3 key findings from this study. First, it is feasible to develop and deploy a real-time resource tool. Second, there was low use of crisis resources overall (including those specifically associated with ResourceBot) in the study. Third, participants rated the ResourceBot with moderate satisfaction and good usability. Each of these findings warrants further comment.

We found that it is possible to build, deploy, and receive high engagement with a real-time crisis resource tool. Much has been written about the promise of smartphone technologies for suicide research and intervention [11,27,28]. Although a plethora of mobile apps exist for suicide prevention [29], little systematic research to date has examined mobile interventions for suicide prevention [30]. This study found that in a severe sample (ie, recent suicidal thoughts, elevated symptoms of depression and anxiety), most of the time participants engaged with the ResourceBot and provided helpful data on their use of the tool. In this paper, we intentionally used a simple decision rule (eg, if suicidal intent is greater than 1 then send ResourceBot) to increase the feasibility and interpretability of findings. To promote greater engagement, future research could increase the complexity in 2 ways. First, the decision rule for the tool could be more adaptive and tailored to the individual, for example, deploying the tool based on participant’s deviations from their own within person average level of suicidal intent or a rule that incorporates additional variables beyond just suicidal intent. Second, a greater number of messages with distinct content could be used in the tool. The barriers and psychoeducation messages were static in this study, which could have resulted in habituation to the ResourceBot and low use of resources. Therefore, a broader more dynamic message base to draw on may promote greater engagement over time in future work. In short, this study found it is feasible to deliver tools for participant safety immediately after participants complete real-time monitoring surveys. Future work can expand upon what type of tools are offered in that immediate moment after a participant has reported suicidal thoughts.

The second finding of this work is that no participants reported using crisis hotlines in the follow-up survey after the ResourceBot. This finding was counter to our expectations given prior work finding that a BRI can increase the self-reported use of crisis resources [9]. Our work highlights how in digital health research one cannot assume findings from one intervention context necessarily translate to another context. There are numerous reasons why there may be differences between past work and this study. This includes differences in the trigger for the BRI and the participants in the studies. For example, prior work was done with naturalistic users of social media platforms and this work was done with EMA study participants. Most participants in this study had a lifetime history of mental health care and many had previously used crisis hotlines. At baseline, participants reported on average feeling unlikely to use crisis hotlines in the future and therefore may have been more resistant to the BRI. Past work has also been with larger samples (ie, hundreds of participants), and we may have seen more participants use hotlines with a larger sample size.

More broadly, given the increasing role of crisis hotlines in national suicide prevention efforts [31] and suicide research safety protocols [5], this study highlights the need to continue to understand participants’ concerns about and experiences with these hotlines. A recent nationally representative survey found that about 5% (23/388) of participants with serious distress had used the 988 Lifeline and only 29% (7/23) of those participants with serious distress reported being very likely to use it in the future [32]. This work suggests the skepticism of crisis hotlines (eg, beliefs that they won’t help) in this study are not unique and perhaps a need to offer a broader range of resources in future work. For example, providing suggestions of coping skills (eg, distraction and relaxation) from interventions such as the safety planning intervention may be incrementally useful [33,34].

Finally, participants rated the ResourceBot with good satisfaction and usability. According to one normative rating scale of the SUS [35,36], the average score in our study for ResourceBot would get a grade of a “B.” According to another rating system [37], it would be considered “good.” These findings provide further support for the feasibility of the real-time deployment of suicide prevention tools. To our knowledge, publicly available norms for the CSQ are not available. Using a transformation suggested by the scale developers to put the score on a 25 to 100 scale where one multiplies the original total score by 3.125, would produce an average score of 65.6 for the ResourceBot. This suggests significant room for improvement with the ResourceBot tool. It is possible that participants’ skepticism of crisis lines influenced their satisfaction with ResourceBot given that the tool promoted the use of these crisis lines. In the future, offering a broader type of message, resources, and skills may increase satisfaction with the tool. Another important finding from the exit survey is that participants reported on average being very comfortable answering questions honestly in the study. Examination of temporal trends in the intent to kill oneself scores also showed no changes in severity by day in the study. If participants were trying to avoid triggering the ResourceBot tool, one may expect to see lower intent scores toward the end of the study period and we do not see this. Both the exit survey honesty ratings and the lack of temporal trends in intent scores, suggest a lack of reactivity to the ResourceBot tool, which is a concern with real-time interventions for suicide prevention [6]. The lack of reactivity could also be due, at least in part, to the anonymous nature of the study [38] and the clear language in the consent form regarding active risk monitoring. The structure of the study and the ResourceBot could have contributed to participants feeling more comfortable disclosing suicidal thoughts [39].

This study provides new information on real-time risk management and crisis resource use but still has important limitations that warrant discussion. First, the current sample was a convenience sample recruited through a web-based research platform. It is unclear how the results would generalize to a clinical sample. Second, the threshold used to deploy the ResourceBot tool was relatively low compared with thresholds used in past research [6]. It is possible that participants did not use the resources because they did not consider their own current suicidal thoughts severe enough to warrant reaching out to a crisis hotline. To try to limit overwhelming participants with the ResourceBot, we implemented a stop rule so that the ResourceBot was only deployed up to once a day. Without this stop rule, the deployment rate would have been 939 rather than 221. Nevertheless, most participants who were sent the ResourceBot in the current study were sent it multiple times. As shown in Figure 4, the average levels of suicidal intent were relatively low, which is one reason why the lower threshold was used. These issues of severity and frequency highlight the challenge of selecting an appropriate threshold of suicidal intent. The engagement with the ResourceBot tool and use of the crisis resources may have been different if a higher threshold was used. Third, the ResourceBot deployment was contingent on compliance with the real-time survey and it is possible that participants may be less likely to fill out a survey when they are experiencing higher levels of distress. More work is needed to better understand compliance in real-time monitoring studies and the best way to incentivize compliance [40]. Fourth, the compliance rate for the exit survey (59.4%) was relatively low, which could have potentially biased the exit survey results. Finally, this study can provide information on the feasibility and usability of automated tools, but cannot fully speak to the ethics of automated interventions versus clinician outreach interventions [4,41]. In this study, the Prolific platform requires that participants maintain anonymity and therefore clinician contact in this setting would not be possible. Decisions related to the type and timing of real-time interventions depend upon the context of the study as well as discussions with ethics boards and regulatory bodies [4,42].

Future studies could build upon this study in multiple ways. First, this study only offered crisis lines as resources. Future work could offer more types of resources in this automated tool format, such as reminders or skills for coping with suicidal thoughts. Second, this study is focused on feasibility and acceptability and no randomization was used. Future work could consider a between-participants randomized control trial where different groups were provided with an automated interactive intervention or pop-up reminders. Future work could also attempt to use a within-person micro-randomized trial design [43] where participants are randomized to different types of automated tools at different levels of suicidal thinking [11]. This type of trial design could allow future studies to empirically test the effect of presenting different kinds of resources on future resource use or self-reported momentary suicidal thinking. Finally, this work highlights the immense complexity and challenge of building ethical and effective real-time interventions for suicide prevention. Future work could use focus groups and other qualitative methods from individuals with lived experience to better understand and develop tools that would be the most helpful to people during moments of elevated suicide risk [44].

Mobile technologies have the potential to advance the understanding of suicide and contribute to new suicide prevention approaches. These technologies, however, present immense ethical challenges in which researchers grapple with both collecting helpful data and preserving participant safety. This study highlights the nuance of this issue and the need for the rigorous development of real-time safety tools.

## Appendix

Multimedia Appendix 1 Images of the ResourceBot and the decision logic and mean (SD) for all items on both scales.

## Abbreviations

BRI: barrier reduction intervention
CSQ: client satisfaction questionnaire
EMA: ecological momentary assessment
GAD-7: Generalized Anxiety Disorder 7-item
PHQ-9: Patient Health Questionnaire-9
SITBI-R: Self-Injurious Thoughts and Behaviors Interview—Revised
STB: suicidal thoughts and behavior
SUS: System Usability Scale
