# Supplementary material for: Automated Real-Time Tool for Promoting Crisis Resource Use for Suicide Risk (ResourceBot): Development and Usability Study
Source: JMIR Ment Health. 2024 Oct 31;11:e58409. doi: 10.2196/58409 (PMC11565085; doi:10.2196/58409)
Supplement: Multimedia Appendix 1 [file mental_v11i1e58409_app1.docx]

**Table S1.** Client Satisfaction Questionnaire Individual Item Scores

| Item Number | Mean (*SD*) |
| --- | --- |
| 1^*^ | 2.8 (1.0) |
| 2 | 2.6 (0.9) |
| 3^*^ | 2.3 (1.0) |
| 4 | 2.8 (0.8) |
| 5 | 2.6 (0.8) |
| 6^*^ | 2.4 (0.6) |
| 7^*^ | 2.7 (0.9) |
| 8 | 2.7 (0.8) |

*Item is reverse scored

**Table S2.** System Usability Scale Individual Item Scores

| Item Number | Mean (*SD*) |
| --- | --- |
| 1* | 2.2 (0.8) |
| 2+ | 3.3 (0.9) |
| 3* | 3.2 (0.9) |
| 4+ | 3.6 (0.8) |
| 5* | 2.5 (1.1) |
| 6+ | 3.4 (0.7) |
| 7* | 3.3 (0.9) |
| 8+ | 3.0 (1.1) |
| 9^*^ | 3.1 (0.9) |
| 10^+^ | 3.2 (1.1) |

*Item is scored by subtracting 1 from the raw item score

+Item is reverse scored

Below we provide the images, text, and decision logic in the ResourceBot tool.

**Slide 1**


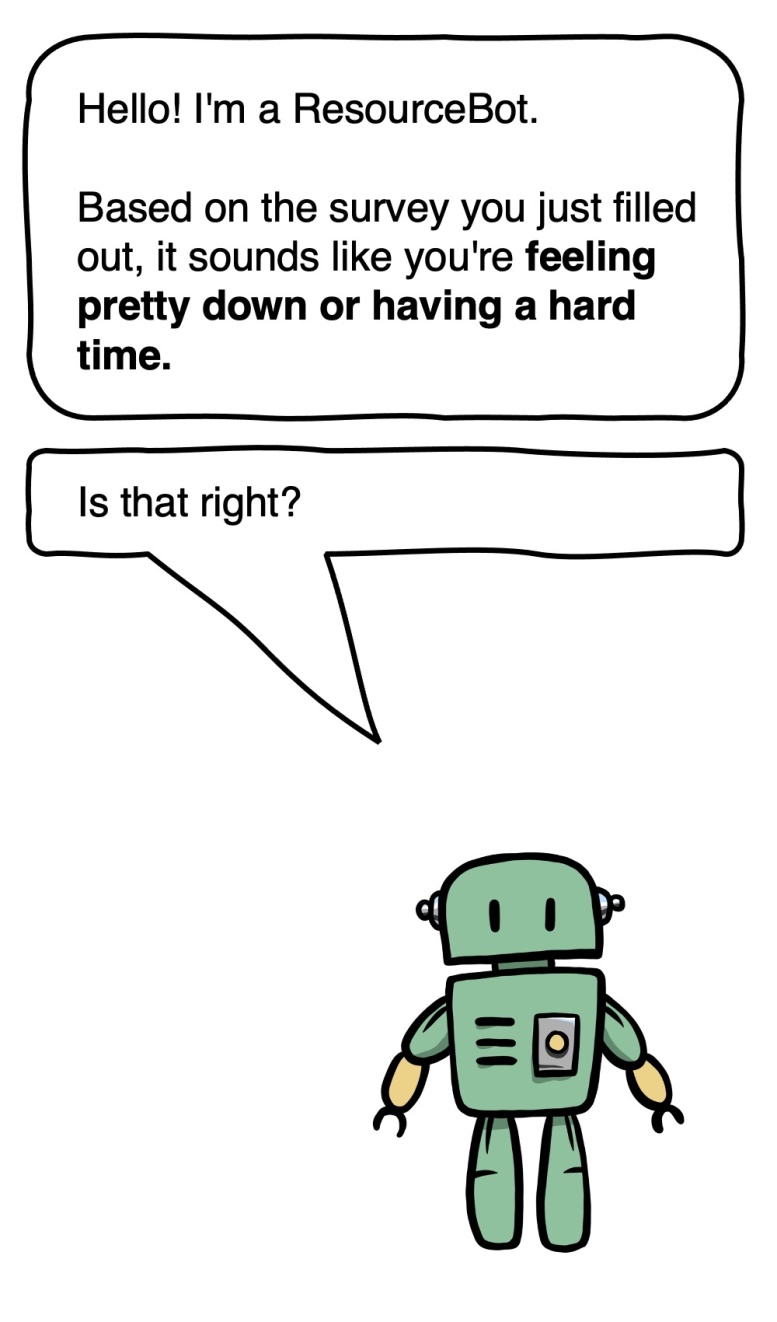


Response Options:

- Yes
- No

**Slide 2**

Shown if “No” on Slide 1


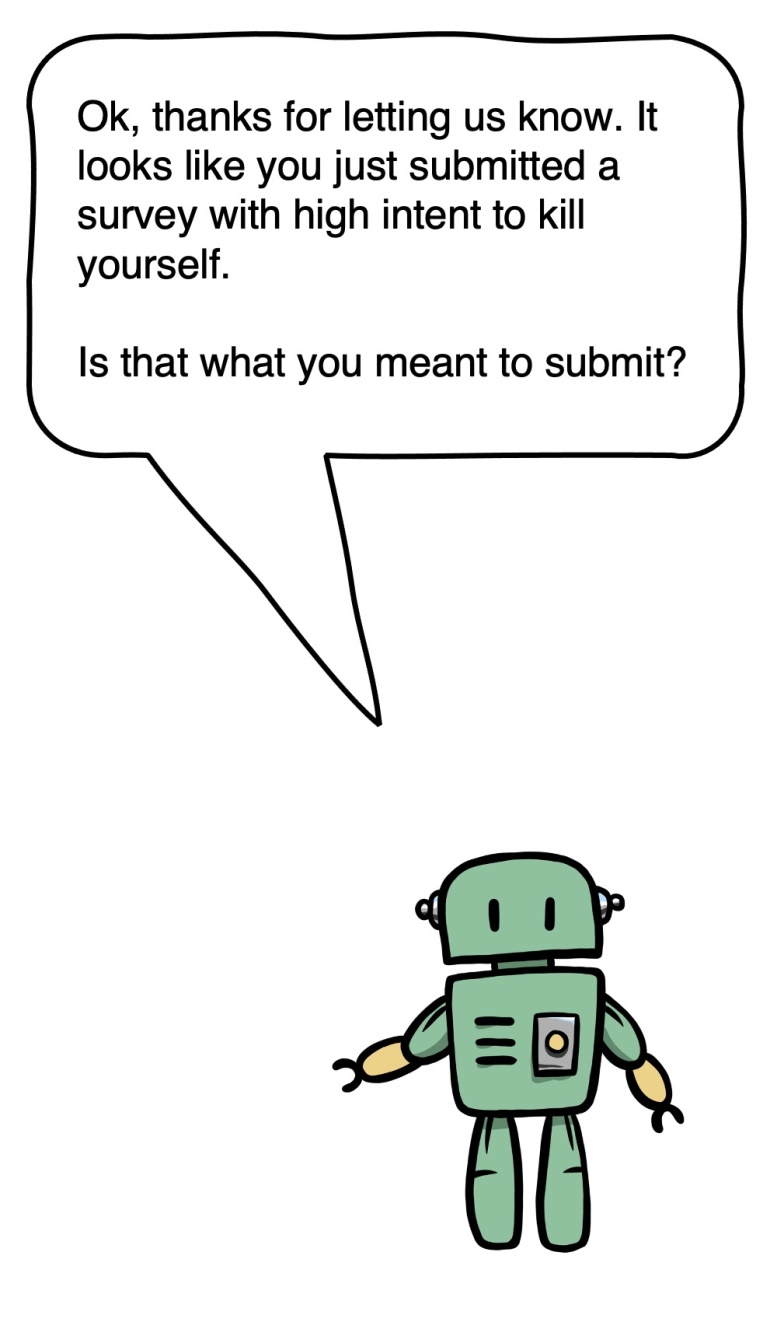


Response Options:

- Yes
- No

**Slide 3**

Shown if “No” on Slide 2


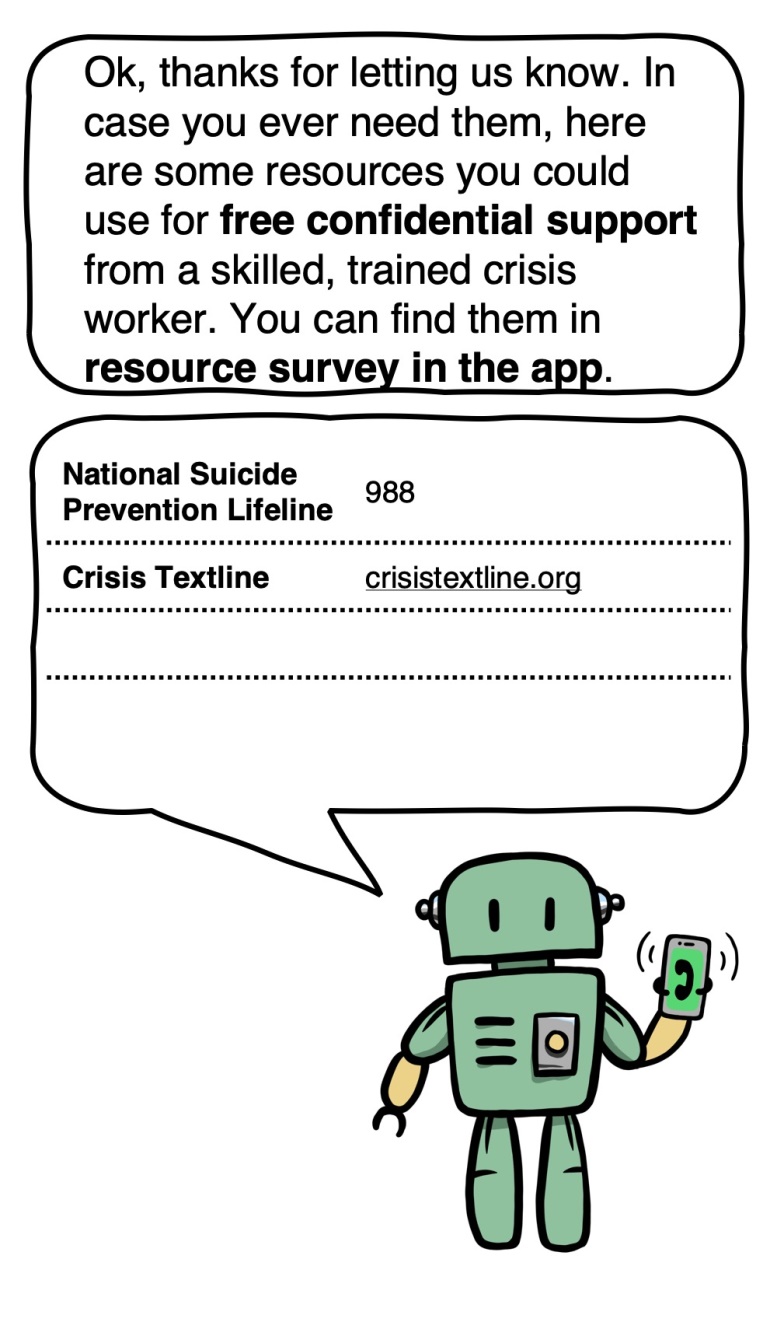


Response Options:

- Ok

**Slide 4**

Shown if “Yes” on Slide 2


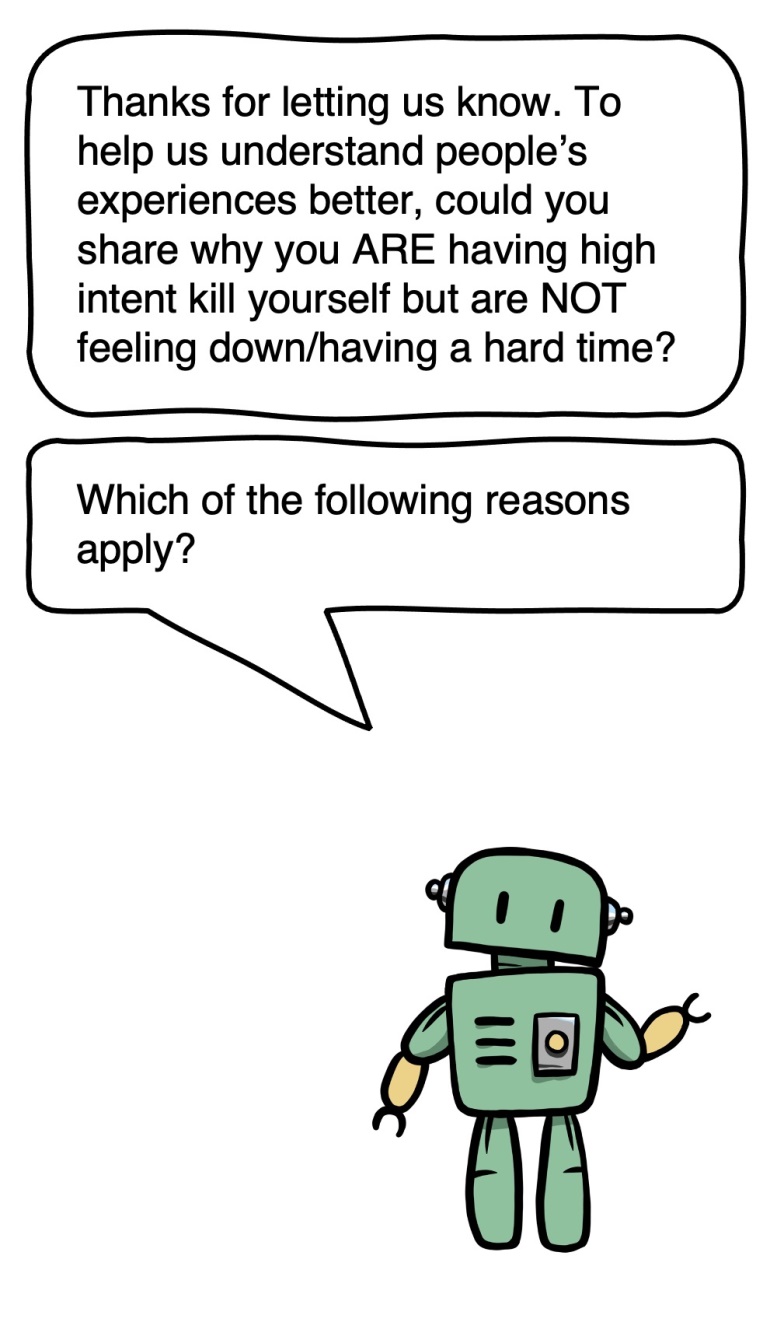


Response Options:

- I am used to these thoughts/feelings.
- I don't need help for these thoughts/feelings.
- Suicidal thoughts help me cope.
- Oops -- I hit the wrong response and want to go back.
- Not really - another reason

**Slide 5**

Shown if “Not really - another reason” on Slide 4


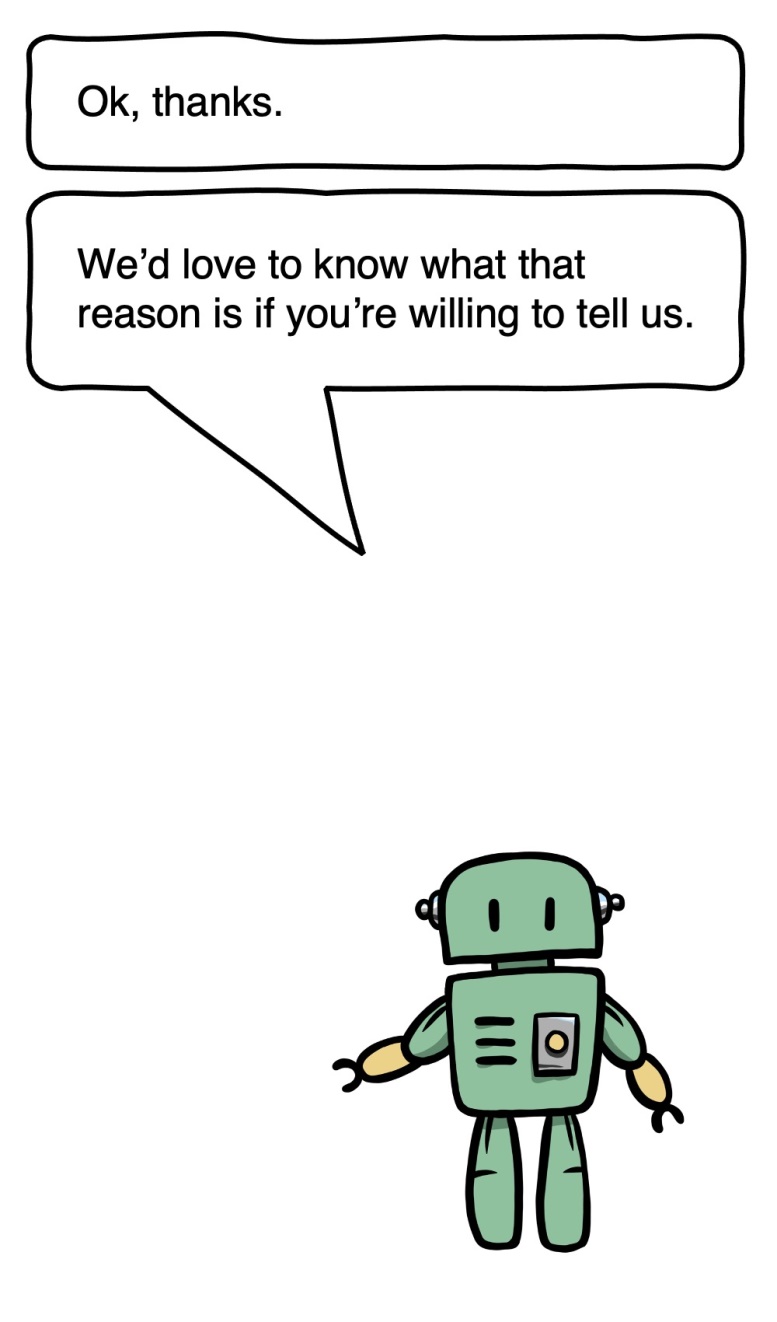


Response Options:

Free Text Entry Box

**Slide 6**

Shown if “I am used to these thoughts/feelings.” or “I don't need help for these thoughts/feelings.” or “Suicidal thoughts help me cope.” on Slide 4


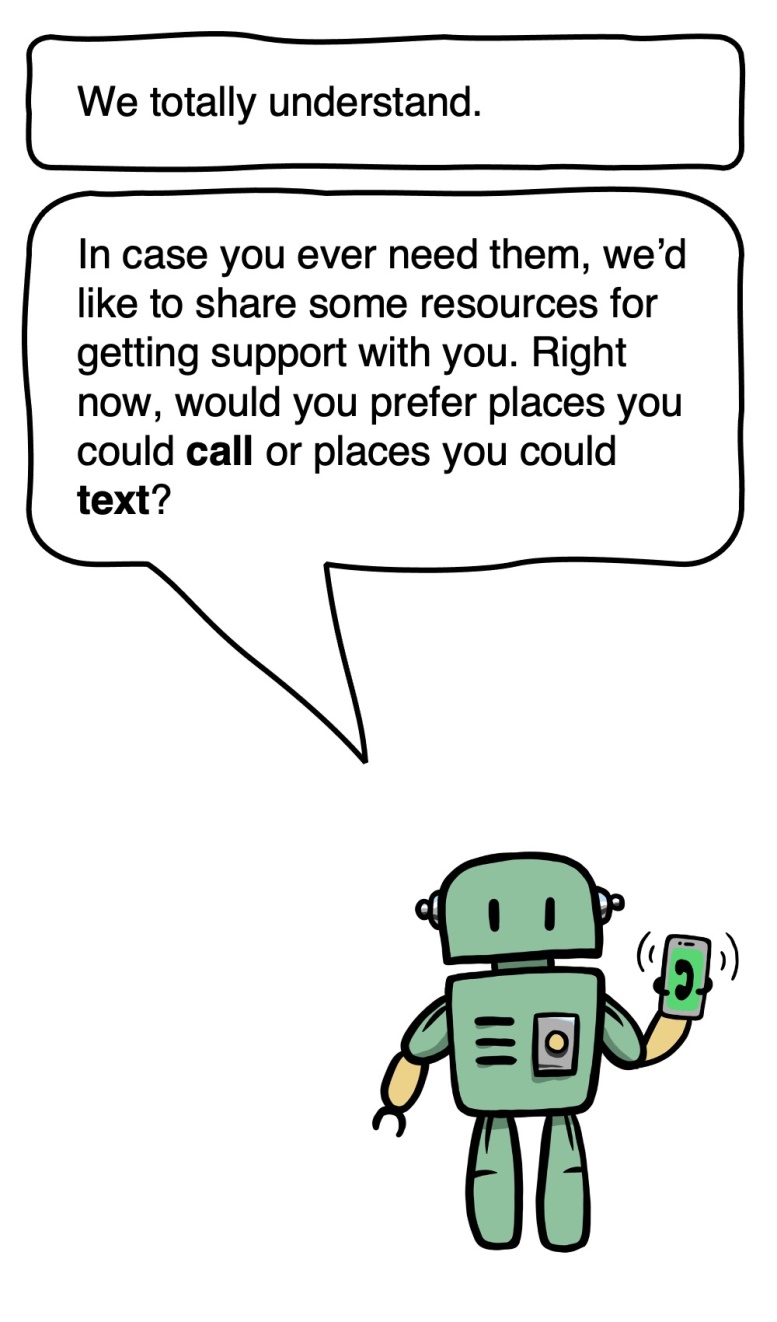


Response Options:

- Call
- Text

**Slide 7**

Shown if Yes on Slide 1


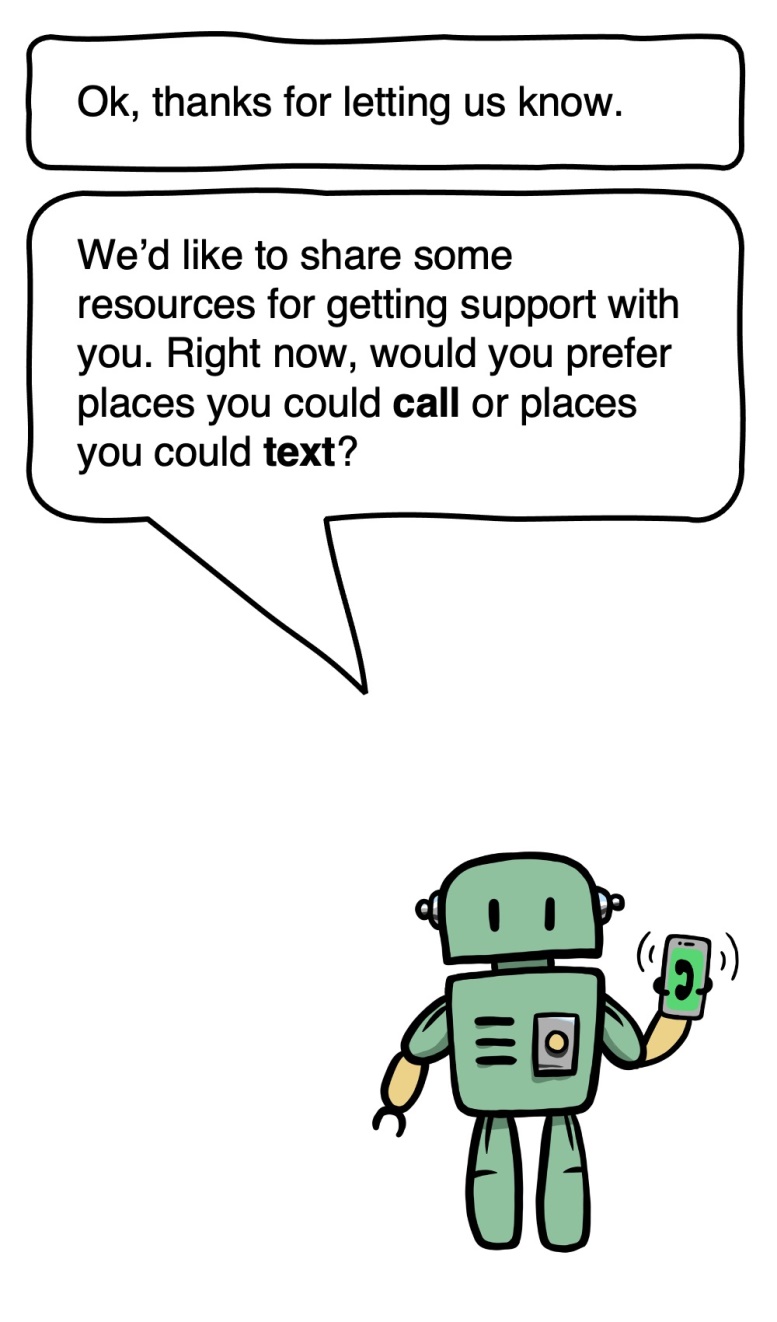


Response Options:

- Call
- Text

**Slide 8**

Shown if Call on Slide 6 or Call on Slide 7


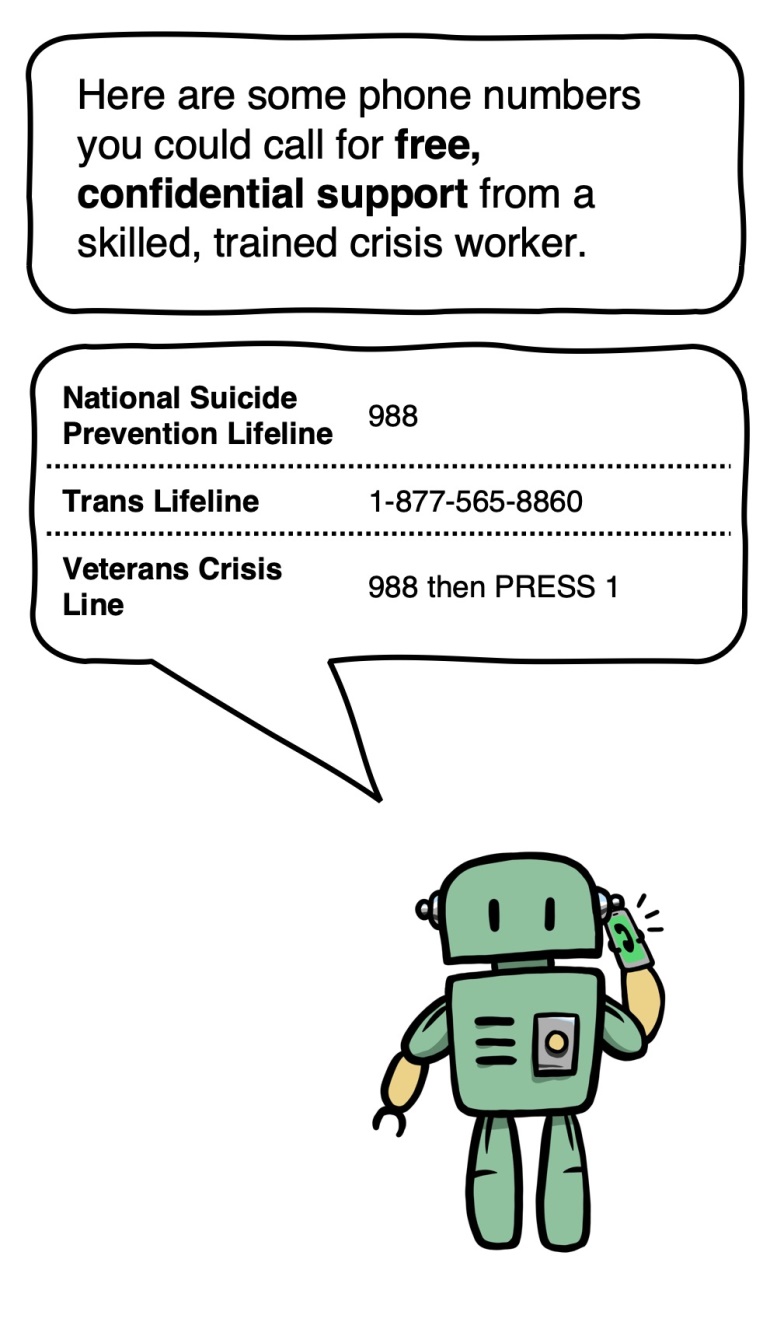


Response Options:

- Ok

**Slide 9**

Shown if Text on Slide 6 or Text on Slide 7


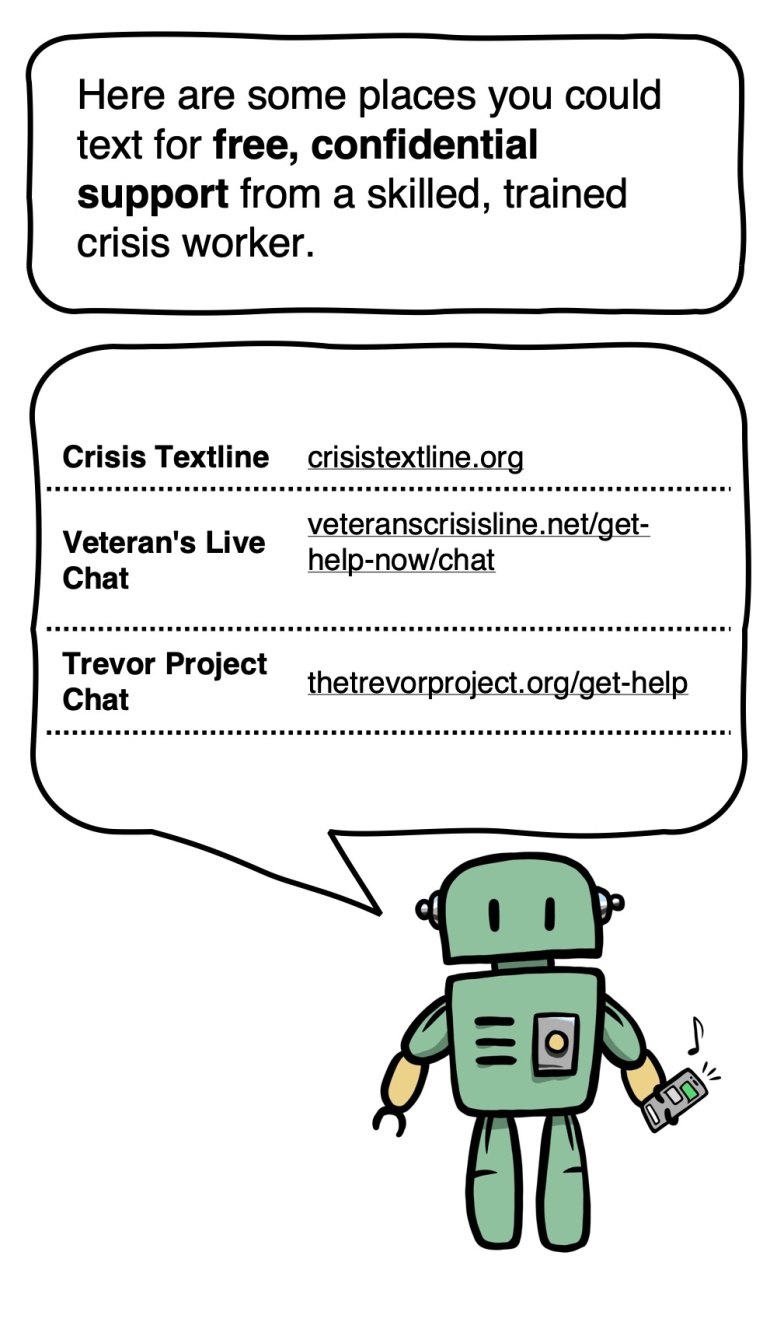


Response Options:

- Ok

**Slide 10**

Shown after Slide 8 or Slide 9


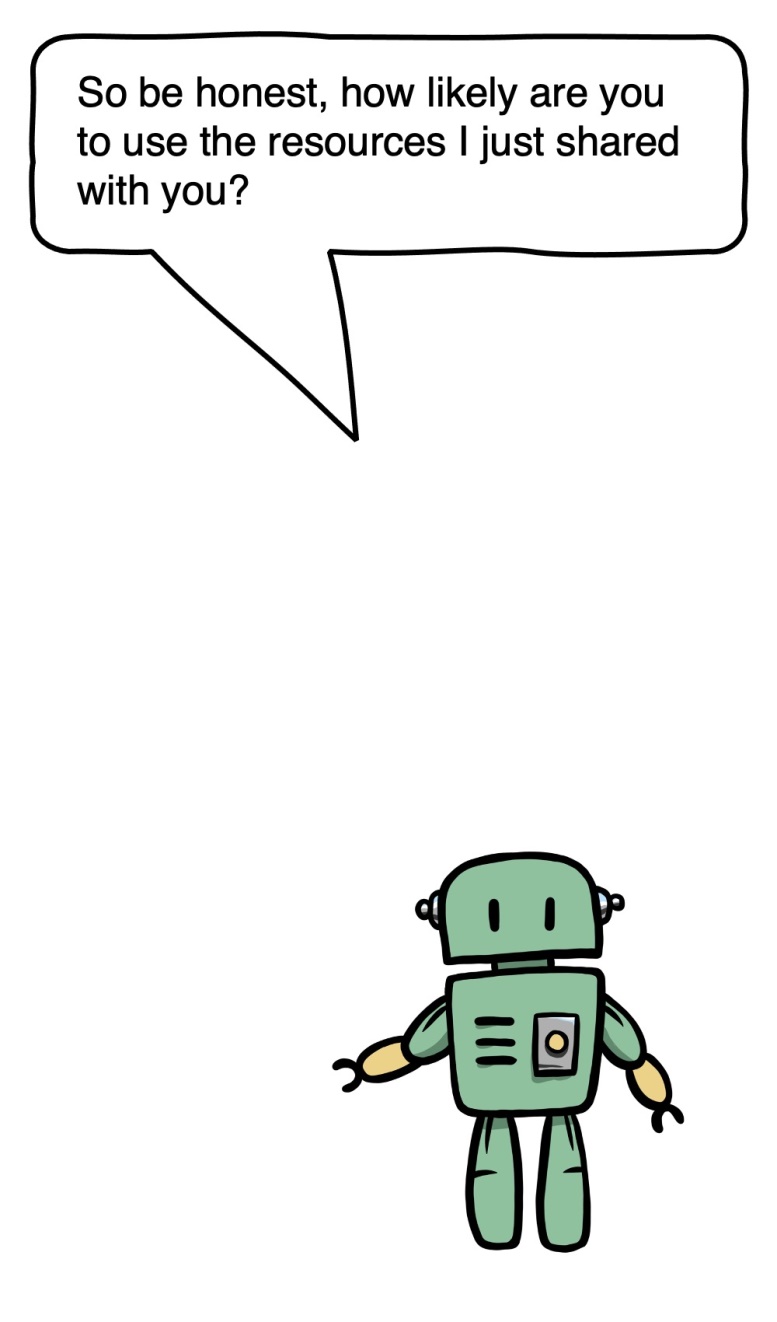


Response Options:

- Not Likely
- Somewhat Likely
- Very Likely

**Slide 11**

Shown if “Not Likely” or “Somewhat Likely” on Slide 10


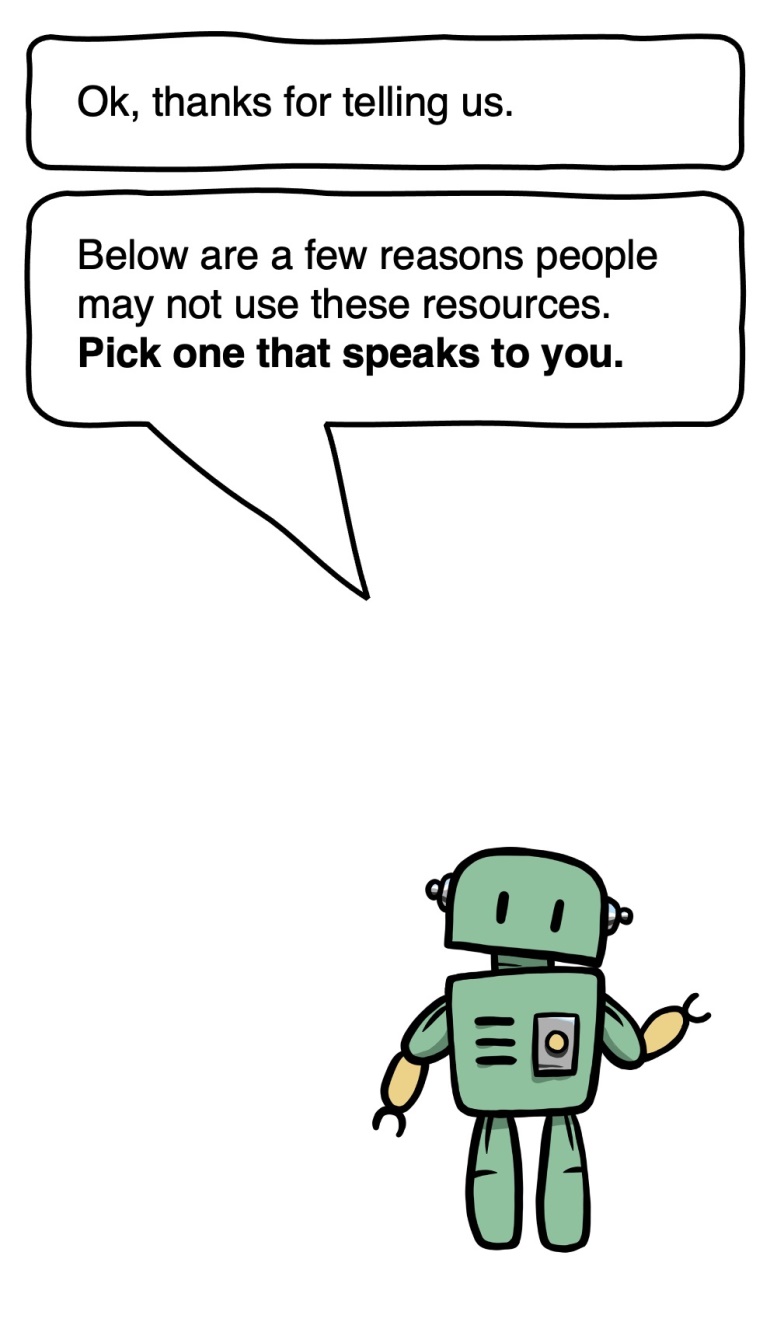


Response Options:

- I can handle it on my own.
- Too much time/effort.
- No professionals.
- No police.
- It won’t help.
- I may not use those resources for a reason not otherwise listed.

**Slide 12**

Shown if “Very Likely” on Slide 10


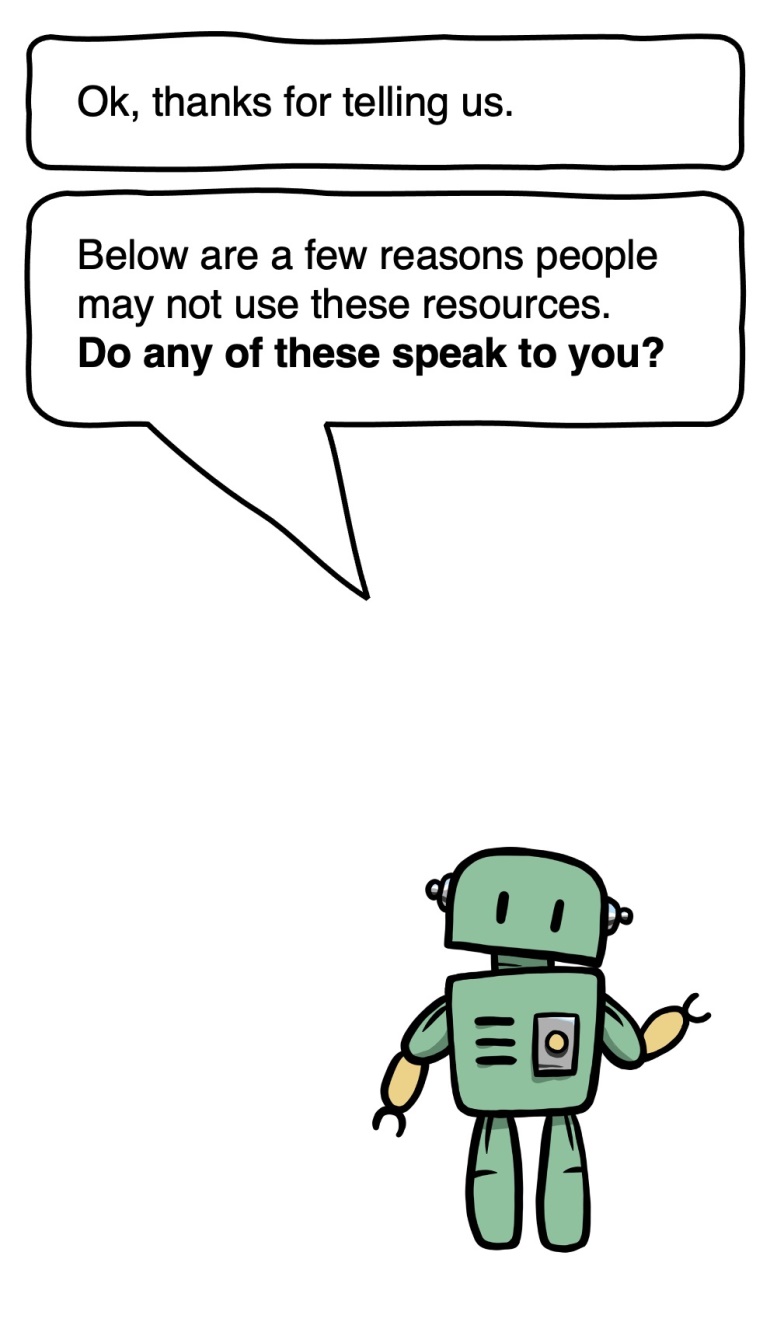


- I can handle it on my own.
- Too much time/effort.
- No professionals.
- No police.
- It won’t help.
- Not really - another reason

**Slide 13**

Shown if “I can handle it on my own” on Slide 11 or “I can handle it on my own” on Slide 12


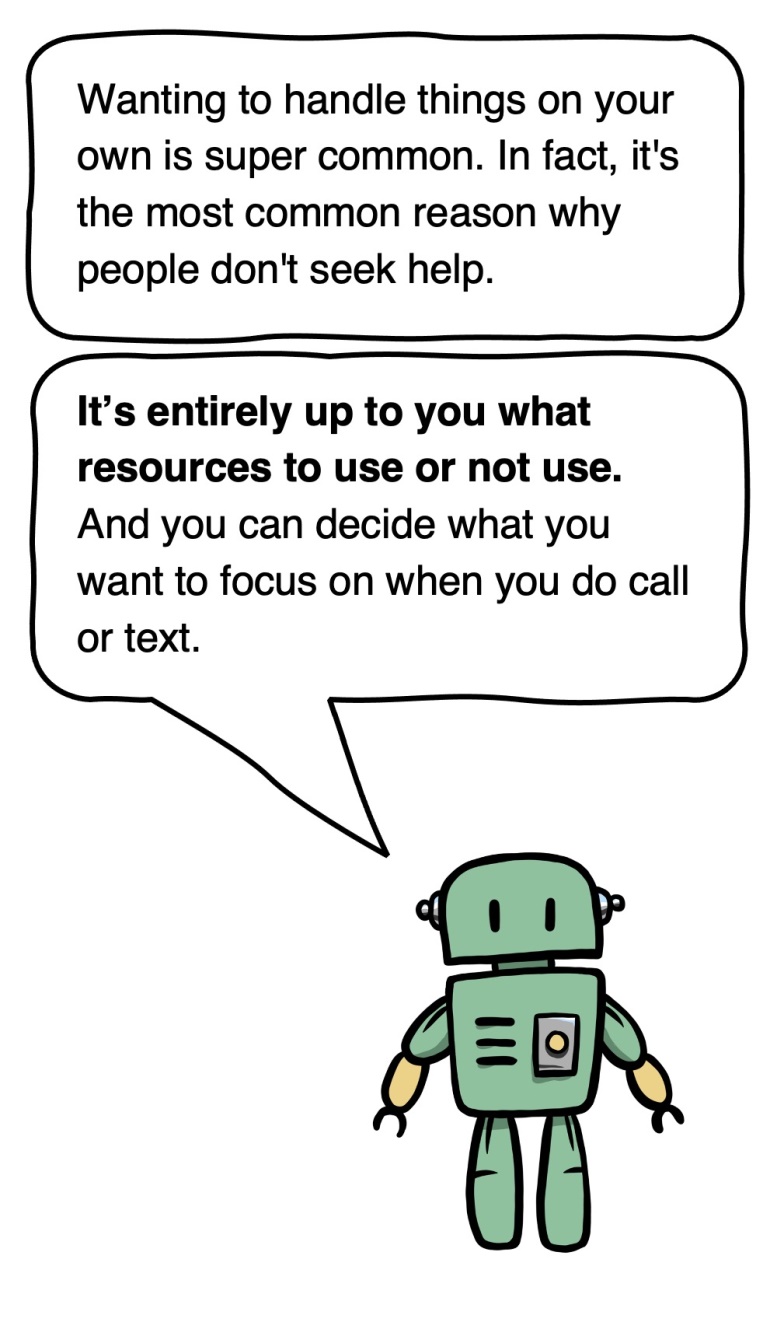


Response Options:

- Ok

**Slide 14**

Shown if “I can handle it on my own” on Slide 11 or “I can handle it on my own” on Slide 12

(Shown after Slide 13)


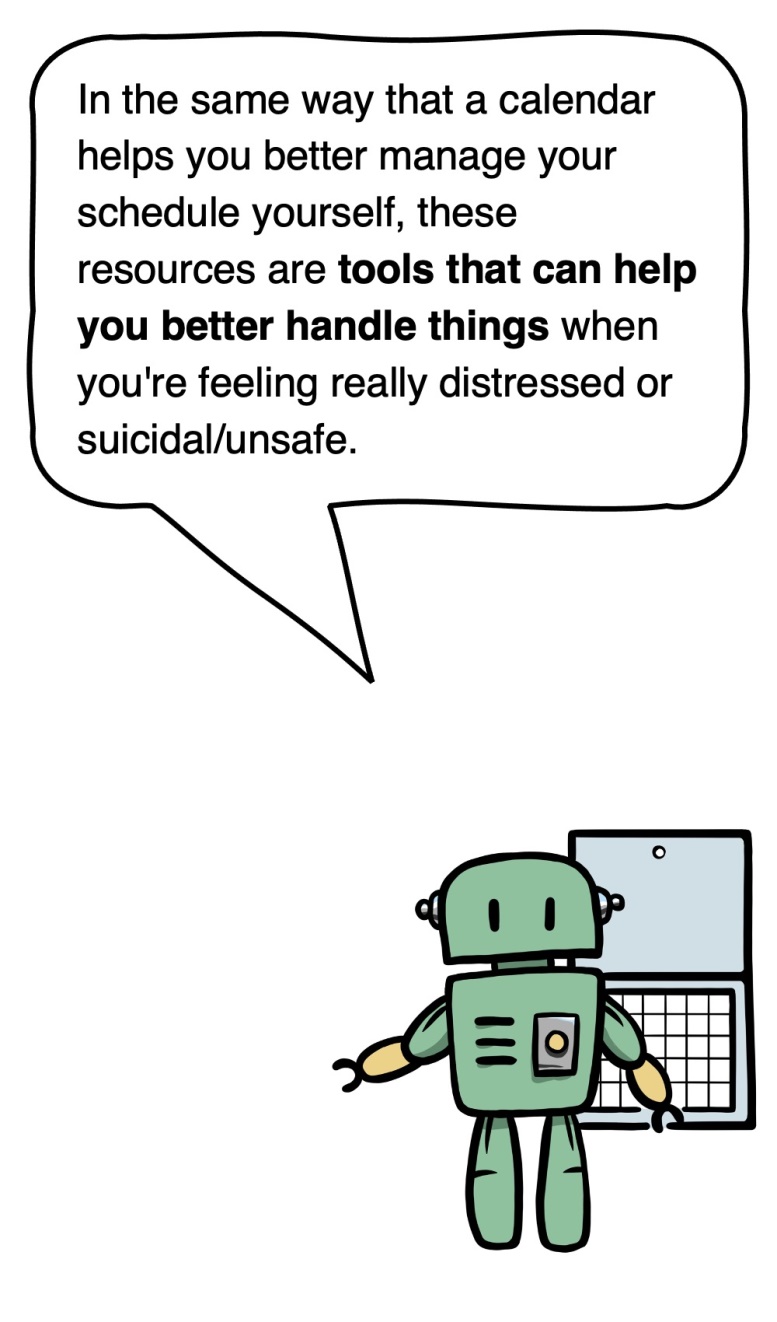


Response Options:

- Ok

**Slide 15**

Shown if “Too much time/effort.” on Slide 11 or “Too much time/effort.” on Slide 12


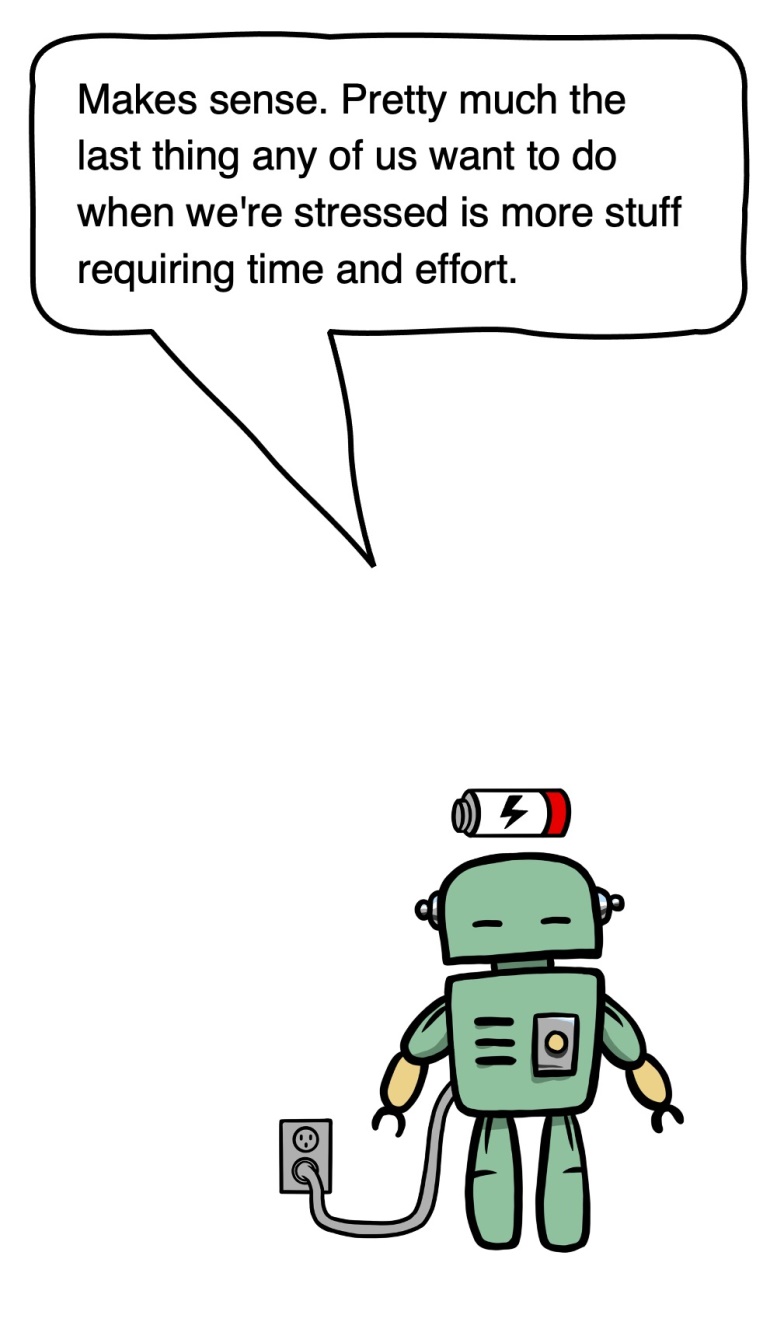


Response Options:

- Ok

**Slide 16**

Shown if “Too much time/effort.” on Slide 11 or “Too much time/effort.” on Slide 12

(After Slide 15)


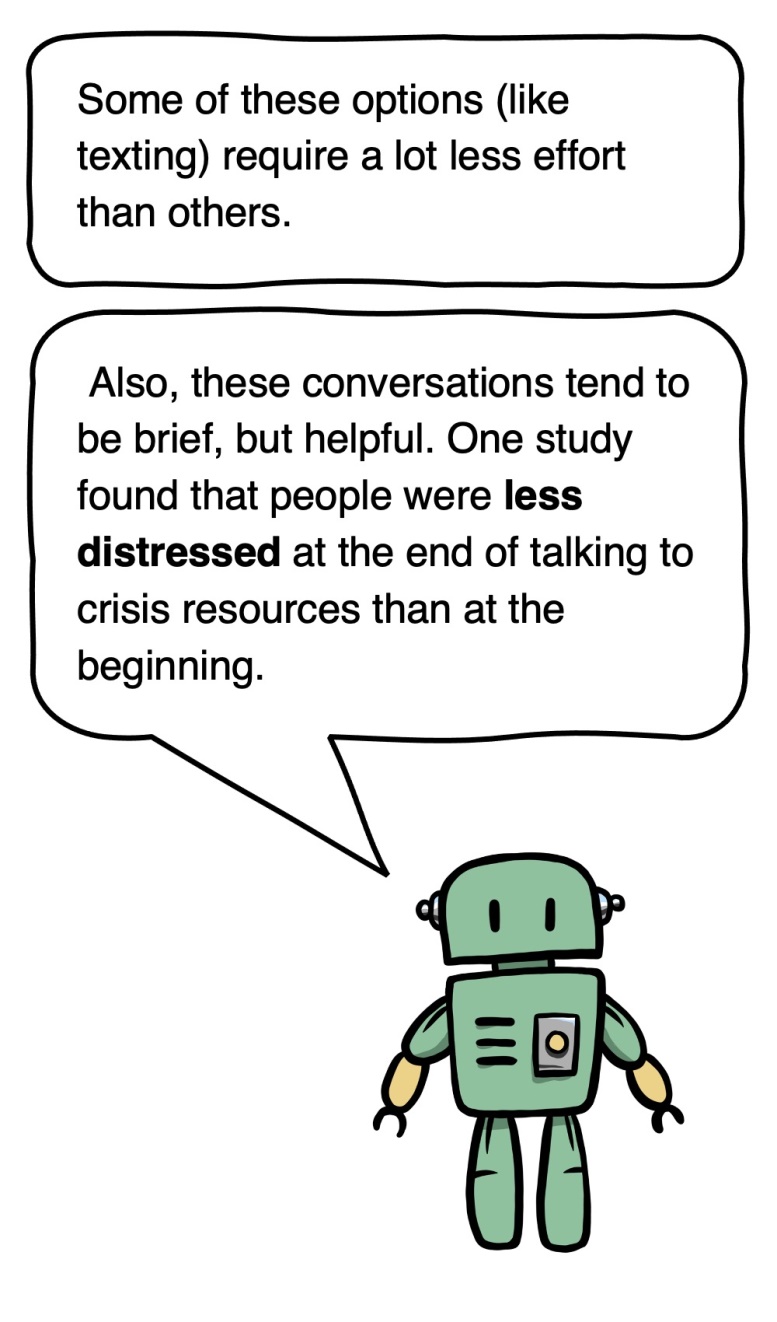


Response Options:

- Ok

**Slide 17**

Shown if “No professionals” on Slide 11 or “No professionals” on Slide 12


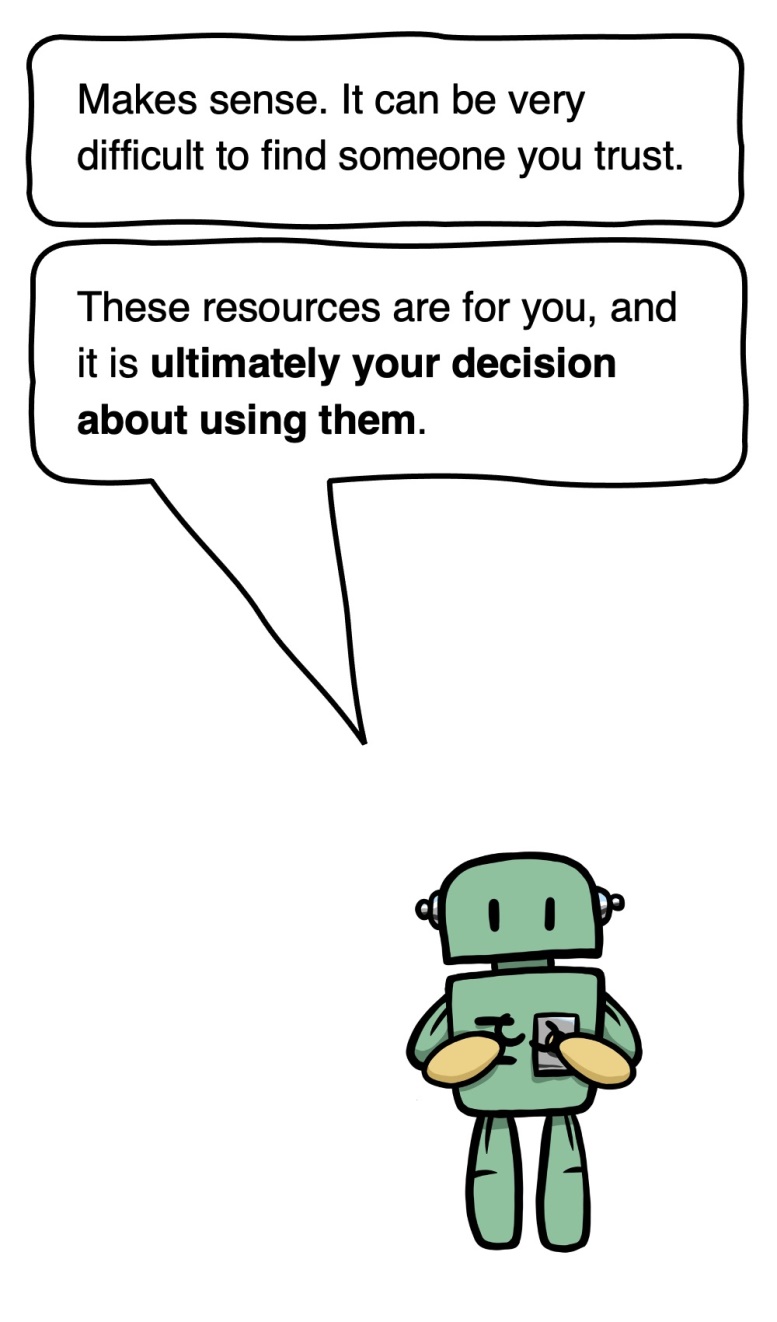


Response Options:

- Ok

**Slide 18**

Shown if “No professionals” on Slide 11 or “No professionals” on Slide 12

(Shown after Slide 17)


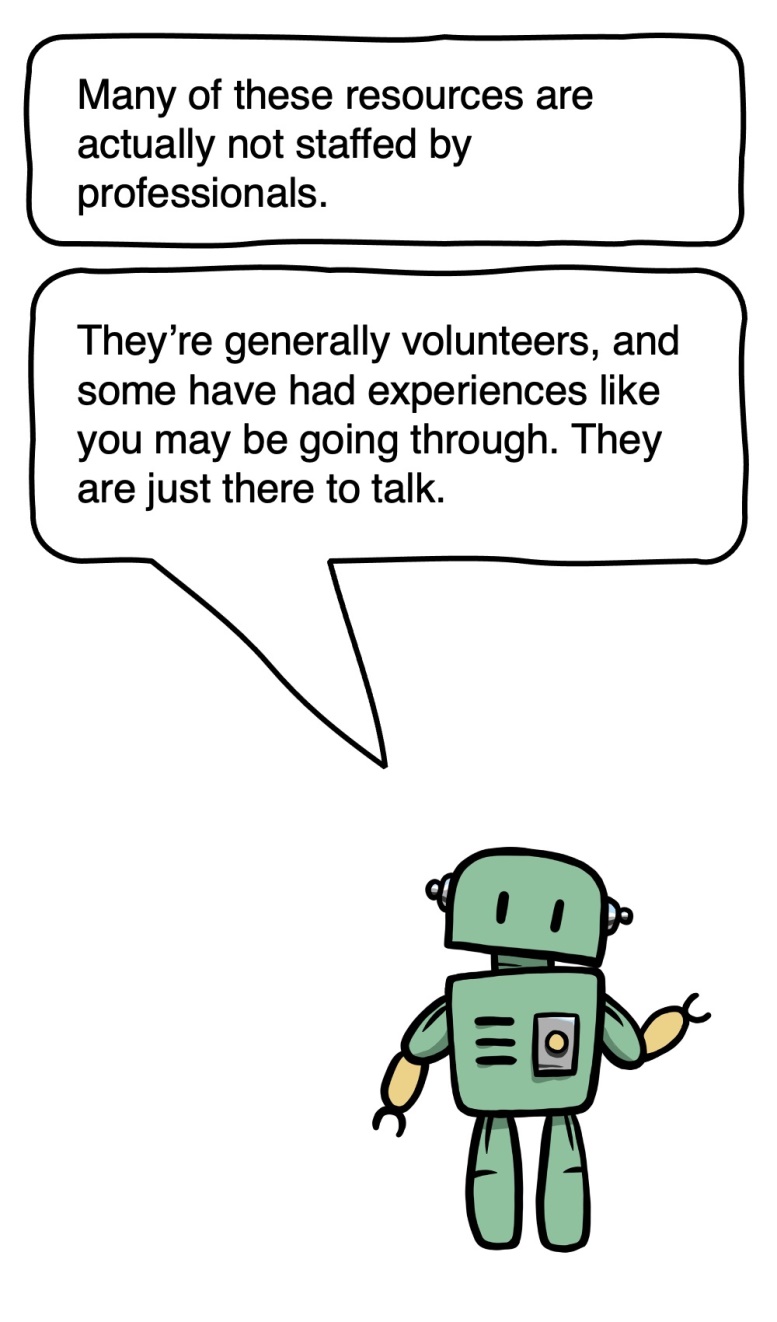


Response Options:

- Ok

**Slide 19**

Shown if “No police” on Slide 11 or “No police” on Slide 12


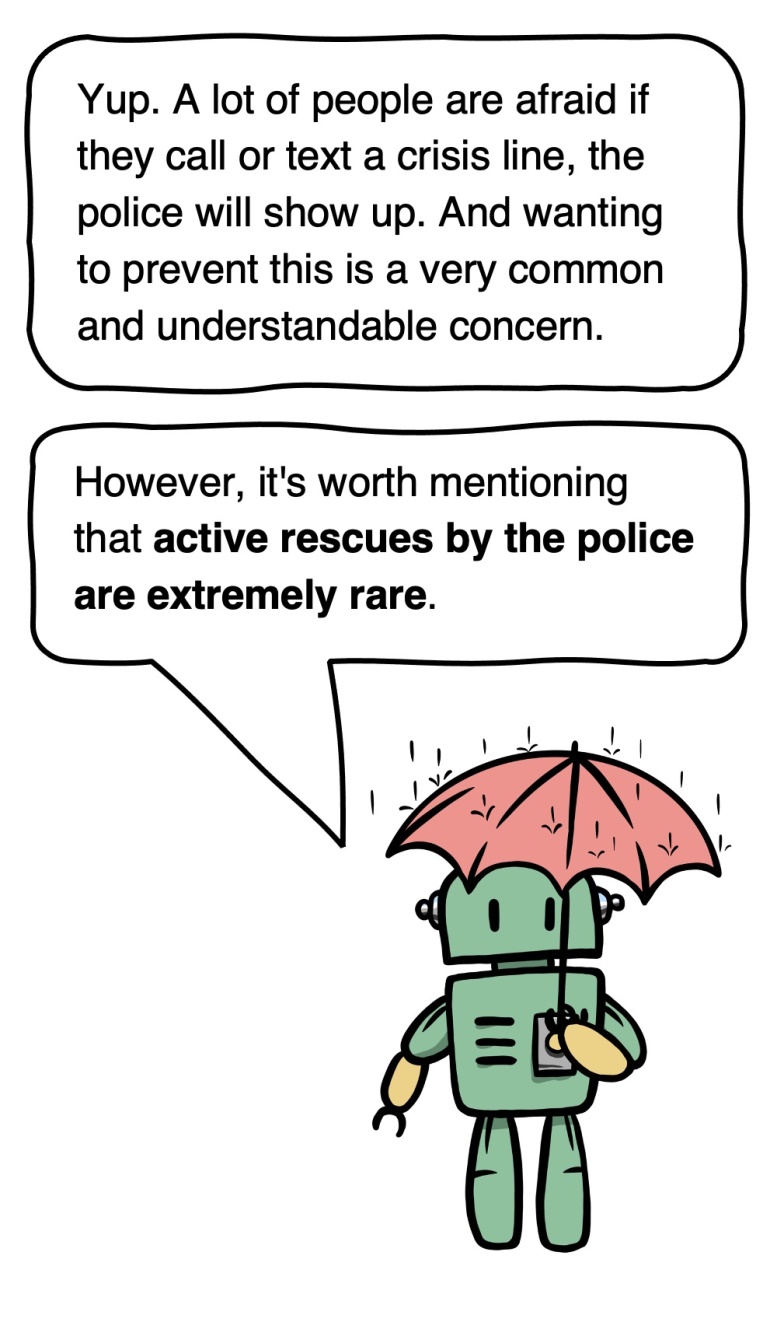


Response Options:

- Ok

**Slide 20**

Shown if “It won’t help.” on Slide 11 or “It won’t help” on Slide 12


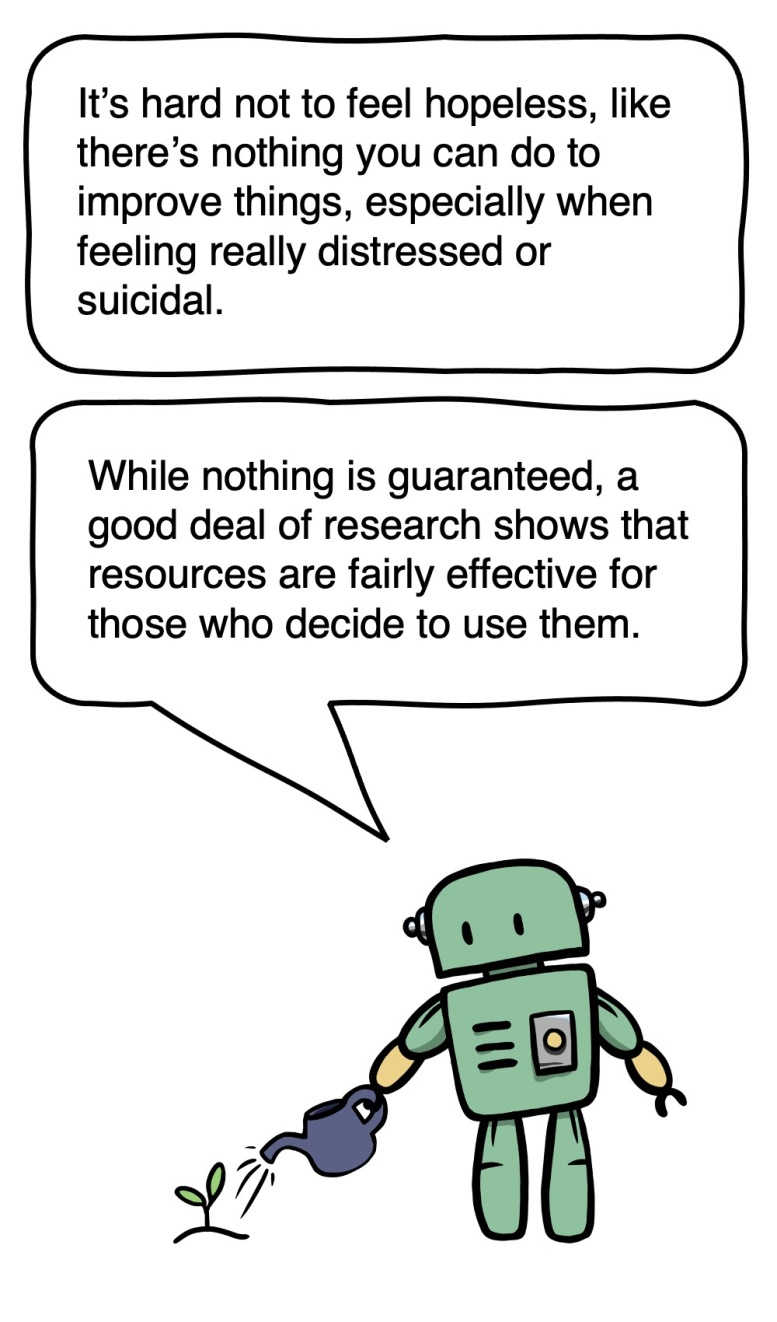


Response Options:

- Ok

**Slide 21**

Shown if “It won’t help.” on Slide 11 or “It won’t help” on Slide 12


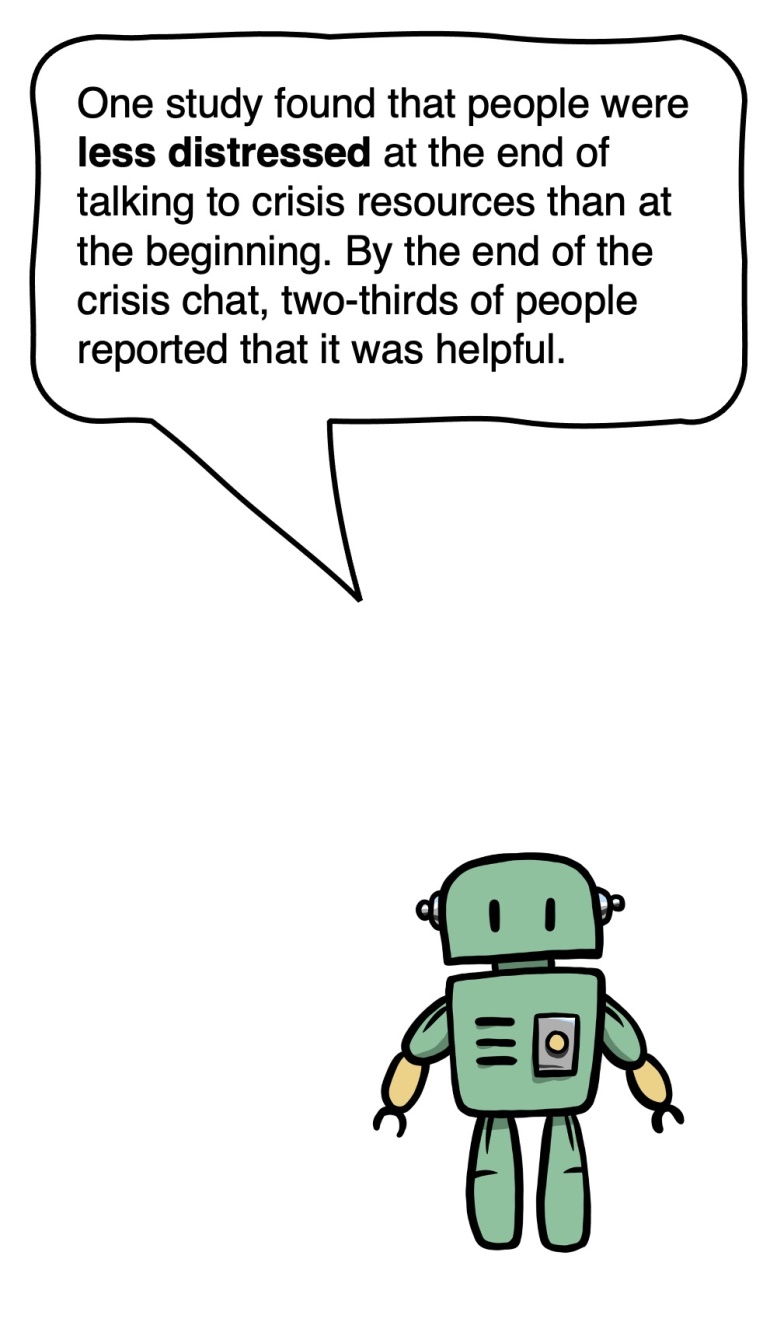


Response Options:

- Ok

**Slide 22**

Shown if “I may not use the resources for a reason not otherwise listed.” On Slide 11 or “Not really - another reason” on Slide 12


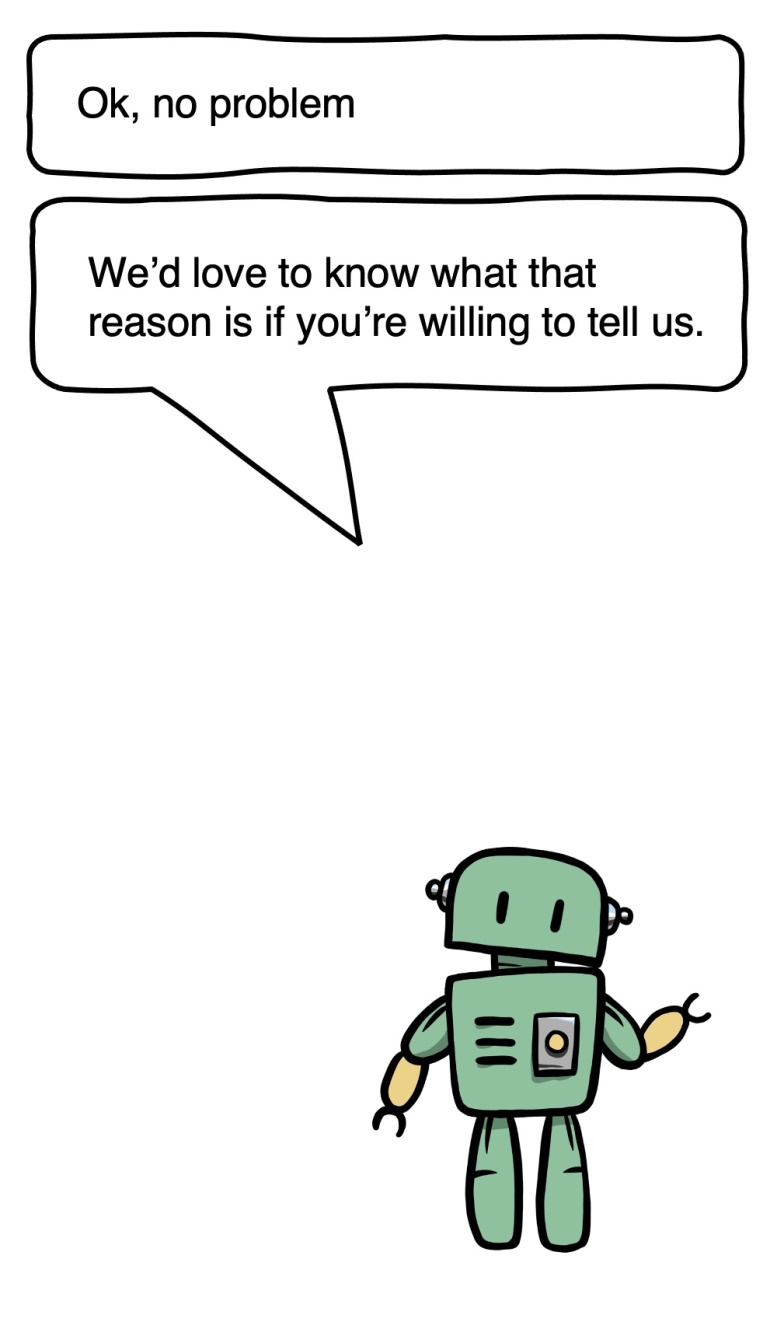


Response Options:

Free text entry

**Slide 23**

(Last slide)


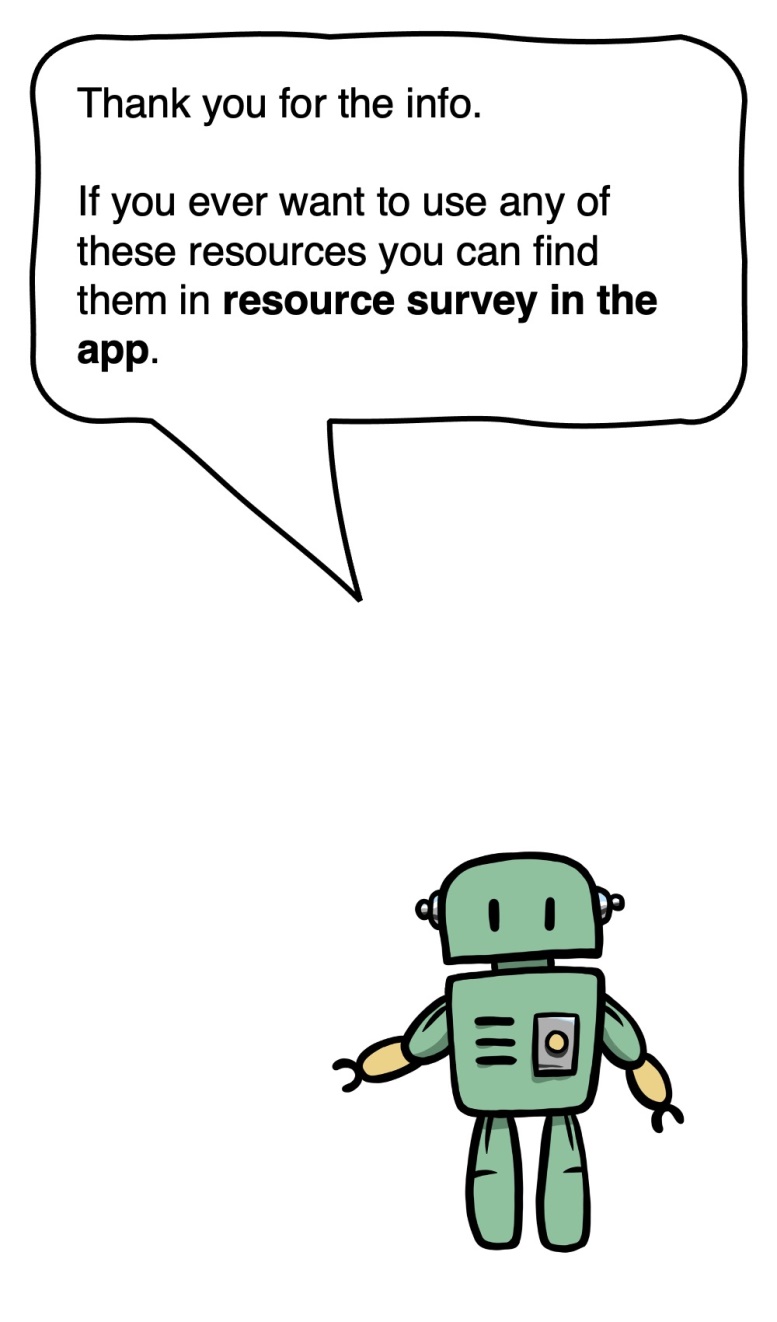


Response Option:

- Ok
